# Monitoring the psychological, social, and economic impact of the COVID‐19 pandemic in the population: Context, design and conduct of the longitudinal COVID‐19 psychological research consortium (C19PRC) study

**DOI:** 10.1002/mpr.1861

**Published:** 2020-11-09

**Authors:** Orla McBride, Jamie Murphy, Mark Shevlin, Jilly Gibson‐Miller, Todd K. Hartman, Philip Hyland, Liat Levita, Liam Mason, Anton P. Martinez, Ryan McKay, Thomas VA Stocks, Kate M. Bennett, Frédérique Vallières, Thanos Karatzias, Carmen Valiente, Carmelo Vazquez, Richard P. Bentall

**Affiliations:** ^1^ Ulster University Coleraine Northern Ireland; ^2^ University of Sheffield Sheffield England; ^3^ Maynooth University Maynooth Republic of Ireland; ^4^ University College London London England; ^5^ Royal Holloway University of London London England; ^6^ University of Liverpool Liverpool England; ^7^ Trinity College Dublin Dublin Republic of Ireland; ^8^ Napier University Edinburgh Scotland; ^9^ Complutense University of Madrid Madrid Spain

**Keywords:** COVID‐19, general population, longitudinal, psychological, survey methodology

## Abstract

**Objectives:**

The C19PRC study aims to assess the impact of the COVID‐19 pandemic in the adult population of the UK, Republic of Ireland, and Spain. This paper describes the conduct of the first two waves of the UK survey (the “parent” strand of the Consortium) during March–April 2020.

**Methods:**

A longitudinal, internet panel survey was designed to assess: (1) COVID‐19 related knowledge, attitudes, and behaviors; (2) the occurrence of common mental health disorders as well as the role of (3) psychological factors and (4) social and political attitudes, in influencing the public's response to the pandemic. Quota sampling (age, sex, and household income) was used to recruit a nationally representative sample of adults.

**Results:**

Two thousand and twenty five adults were recruited at baseline, and 1406 were followed‐up one‐month later (69.4% retention rate). The baseline sample was representative of the UK population in relation to economic activity, ethnicity, and household composition. Attrition was predicted by key socio‐demographic characteristics, and an inverse probability weighting procedure was employed to ensure the follow‐up sample was representative of the baseline sample.

**Conclusion:**

The C19PRC study data has strong generalizability to facilitate and stimulate interdisciplinary research on important public health questions relating to the COVID‐19 pandemic.

## 1 INTRODUCTION

Without an effective therapeutic or vaccine aided prophylaxis for the SARS CoV‐2 virus (hereafter, COVID‐19), governments around the world have imposed stringent social distancing measures to slow contagion, protect the most vulnerable, and better manage health care service demand/provision (World Health Organisation, 2020a, 2020b). The implementation of such “lockdown” strategies, however, has in turn, unsurprisingly, impacted upon and compromised many facets of social and economic life, resulted in mass unemployment, and sparked fears of an impending global economic crisis and recession (Anderson, Heesterbeek, Klinkenberg, & Hollingsworth, 2020; Hale, Petherick, Phillips, & Webster, 2020). Although the extant literature on the non‐biological sequalae of outbreaks of other infectious respiratory diseases (IRDs), specifically SARS‐CoV (SARS), the H1N1 flu pandemic, and the Middle East respiratory syndrome (MERS), provides a useful “roadmap” for COVID‐19 related research, the unique magnitude of this pandemic, that varies considerably from one country to another (and even across regions within a country), means that the psychological, social, and economic impact of the pandemic is largely unknown.

In March 2020, the COVID‐19 Psychological research consortium (C19PRC) was formed to address an urgent need to conduct timely, high‐quality research to generate a robust evidence base that could be used by policymakers and clinicians to successfully navigate the rapidly evolving COVID‐19 crisis. The C19PRC comprises psychologists, mental health researchers, and political scientists, and involvement from non‐academic stakeholders in the disciplines of public health and social care, from across the UK, the Republic of Ireland, and Spain. The C19PRC's chief aim is to monitor and assess the long‐term psychological, social, and economic impact of the pandemic, and to investigate how this might vary across countries that differed in relation to their public health response strategies. Two core principles directed that research conducted by the C19PRC would be: (1) informed by previous studies that investigated the non‐biological consequences of previous outbreaks of IRDs and on recommendations from researchers and public health representatives in response to these outbreaks; and (2) responsive to the unique socio‐political‐economic landscape of the countries involved, and to prioritize the exploration of between‐country factors that might help explain differences in COVID‐19 related outcomes. What follows is an overview of the preparatory work conducted to devise the C19PRC's research methods framework.

### 1.1 Principle 1a. C19PRC‐ Who to study?

Studies investigating the psychological impacts of SARS, H1N1, and MERS predominantly focused on health care workers and patients (SARS [Chong et al., 2004; A. M. Lee et al., 2007; Maunder et al., 2003; Tam, Pang, Lam, & Chiu, 2004; Wu et al., 2009]; H1N1 [Goulia, Mantas, Dimitroula, Mantis, & Hyphantis, 2010; Matsuishi et al., 2012]; MERS [Bukhari et al., 2016; Shin et al., 2019; Um, Kim, Lee, & Lee, 2017]). These studies resoundingly showed that those who provisioned or were in receipt of health care during these crizes were at significant increased risk for an array of mental health problems including anxiety, depression, and traumatic stress. In some countries, for some individuals, the psychological impact of these viruses was suggested to have been greater than the physical health danger posed by the diseases themselves (Cheng & Tang, 2004) and, in the case of SARS, multiple studies referred to this particular outbreak in terms of a “mental health catastrophe” (Gardner & Moallef, 2015; Mak, Law, Woo, Cheung, & Lee, 2009). However, although fewer in number, an array of studies also investigated the psychological impacts of these IRDs among general population samples (SARS [J. T. Lau et al., 2005; Leung et al., 2003; Mak et al., 2009; Zhu, Wu, Miao, & Li, 2008]; H1N1 [Cowling et al., 2010; Jones & Salathe, 2009; Liao, Cowling, Lam, Ng, & Fielding, 2014; Wong & Sam, 2011]; MERS [Batawi et al., 2019] and specific subgroups of the population [e.g. women in midlife [Yu, Ho, So, & Lo, 2005]; pregnant women [D. T. Lee et al., 2006; Ng, Sham, Tang, & Fung, 2004]; elderly and younger people [A. L. Lau et al., 2008]). These studies revealed complex, nuanced, and often severe psychological consequences of IRDs that extended beyond the “frontline” impacts of virus detection, treatment, and recovery.

Unsurprisingly, the National Advisory Committee on SARS and Public Health (Naylor et al., 2003) proposed that a “systemic perspective” was needed and should be prioritized by those engaging in IRD psychosocial research. This recommendation suggested that psychosocial research should not be restricted to health care workers and patients during such crizes and that populations such as nonmedical personnel and the public should also be assessed. Such an approach would enable more comprehensive and balanced planning of efforts to alleviate the psychosocial burden of IRDs in the population at large or mitigate its onset in the future (Sim & Chua, 2004).

### 1.2 Principle 1b. C19PRC: When to study?

A second recommendation proposed by the National Advisory Committee on SARS and Public Health (Naylor et al., 2003) was that prospective research should be prioritized because the psychological impact of IRDs may persist or evolve over time. For example, several studies that have investigated the psychological impacts of SARS, MERS, and H1N1 have revealed the ongoing, and in some cases, worsening psychological effects over periods of months and years post outbreak (Cowling et al., 2010; Gardner & Moallef, 2015; Shin et al., 2019). Longitudinal studies facilitate assessment of the important determinants of, and changes in, psychological distress as well as the protective effects of certain coping strategies and behaviors during critical periods of IRD outbreaks (i.e., from first case to peak death rate to societal recovery).

### 1.3 Principle 1c. C19PRC: What to study?

The extant evidence base on outbreaks of IRDs also provided valuable direction in relation to a variety of other relevant issues that warrant investigation during the pandemic. Significant attention has also been paid to the role, context and change in public health knowledge, attitudes, behaviors, and practices over the course of IRD outbreaks (Alsahafi & Cheng, 2016; Cowling et al., 2010; Karademas, Bati, Karkania, Georgiou, & Sofokleous, 2013; J. Lau, Griffiths, Au, & Choi, 2011; J. T. Lau, Griffiths, Choi, & Tsui, 2010; Lin et al., 2011). Some investigators have focused specifically on risk perceptions during IRD epidemics/pandemics (Cho & Lee, 2015; Ibuka, Chapman, Meyers, Li, & Galvani, 2010; Shi et al., 2003; R. D. Smith, 2006), while others have investigated the occurrence and consequences of phenomena such as paranoia (Cheng, 2004), uncertainty (Taha, Matheson, & Anisman, 2014), rumor, and superstitious beliefs (Tai & Sun, 2011). A specific literature (common to H1N1 and MERS) has addressed the role and impact of social media and news broadcasting during these outbreaks (H1N1 [McNeill, Harris, & Briggs, 2016; Taha, Matheson, & Anisman, 2013; Tausczik, Faasse, Pennebaker, & Petrie, 2012; Wong & Sam, 2010b]; MERS [Choi, Yoo, Noh, & Park, 2017; Lim, Lee, Kim, & Chang, 2017; Ludolph, Schulz, & Chen, 2018; Seo, 2019; Yoo, Choi, & Park, 2016]). Factors influencing the uptake of vaccination, decision making and intentionality regarding vaccine use, and parental consent regarding vaccination of children have also received much attention (Brown et al., 2010; Byrne, Walsh, Kola, & Sarma, 2012; Cole et al., 2015; McNeill et al., 2016; Wong & Sam, 2010a). This body of research captures a variety of issues such as the role of trust in the media in determining vaccination intentions, public anxiety associated with information seeking, general health information dissemination, and the effects of mass media exposure on the uptake of preventive measures by the public.

### 1.4 Principle 2. C19PRC: Prioritizing a multi‐country approach to researching the impact of the pandemic.

Between‐country differences in COVID‐19 related outcomes, although clearly evident in the earliest surveillance studies (World Health Organisation, 2020a, 2020b), are not easily explained, and may be related to a host of macro and micro‐level factors including: (1) the size and geographical location of a country; (2) the characteristics of a country's population (e.g. age/sex/health conditions distribution); (3) the level of population density within a country; (4) the ability of a country's health service to conduct widespread testing for COVID‐19, to initiate and maintain a robust contact‐tracing system for confirmed COVID‐19 cases, and to provide optimal care for individuals diagnosed with COVID‐19; (5) the timing, nature and severity of “lockdown” restrictions (and the subsequent implementation of policies and procedures to “unwind” such restrictions); and (6) the public's motivation and ability to comply with the lockdown restrictions, as well as their attendance to, and engagement with, national public health initiatives enacted to prevent the spread of the virus (Hale et al., 2020, p. 31; V. J. Lee, Chiew, & Khong, 2020; Olagnier & Mogensen, 2020; Wyper et al., 2020).

### 1.5 C19PRC objectives

Armed by the research evidence underpinning the C19PRC's two core principles, five objectives were set for the Consortium's research methods framework: (1) to recruit a large, nationally representative panel of adults in each country, that will serve as a study “spine” in that country, and that will facilitate various opportunities for conducting bespoke studies targeting specific sub‐groups in the population (e.g. frontline workers) who are particularly at risk for COVID‐19; (2) to collect detailed “baseline” survey data on a broad range of outcomes and behaviors, such as mental health disorders (e.g., anxiety, depression) and health‐related behaviors (e.g., maintaining hygiene practices, face‐mask wearing), known to be impacted by, and influence recovery from, a global pandemic; (3) to assess a broad array of protective and risk factors known to (or thought to) influence identified health‐related outcomes and behaviors, both at the micro‐level (i.e., via respondent self‐report) and macro‐level (i.e., via linkage of respondent geospatial data to external data resources e.g. population density; availability of green spaces; area‐level rates of COVID‐19 infection, etc.); (4) to re‐contact respondents regularly as the pandemic unfolds, with measurements at each assessment being guided by both the extant literature on previous pandemics, but also adapting and responding to, the unfolding social, political, and economic circumstances in each country; and (5) to produce rapid, high‐quality, country‐specific research outputs in the first instance, for the purpose of contributing to the emerging evidence base pertaining to the pandemic at a national level, and also, to prioritize multi‐country research outputs to highlight, more broadly, whether and how between‐country differences might uniquely explain variation in COVID‐19 outcomes.

The C19PRC prioritized consistency in the measurement of core study outcomes (e.g. mental health disorders; engagement in COVID‐19 health‐related behaviors) across study countries, collection of baseline data at the earliest opportunity in each country, and also timely, roughly equal follow‐up data collection in each country. Flexibility to tailor sections of the survey to address and assess country specific issues (e.g. differences in “lockdown” strategies) was also recognized as an important feature of the study.

The C19PRC study was designed as an internet‐based nationally representative panel survey. The first C19PRC survey commenced in the UK in mid‐March 2020, 52 days after the first case of COVID‐19 had been confirmed in the UK. The first wave of this UK survey (hereafter, C19PRC‐UKW1) is the “parent” survey of the C19PRC Study; subsequent rollouts in other countries (so far, the Republic of Ireland and Spain–both of which commenced in late‐March/early April 2020), were modeled on the design of the C19PRC‐UKW1. The remainder of this paper describes the content and conduct of the C19PRC‐UKW1 and the follow‐up survey at wave 2 (hereafter, C19PRC‐UKW2). While the paper serves as a broad methodological overview for the first and follow‐up surveys in all three countries, minor differences in methodologies across countries will be described in country‐specific papers (e.g. see Hyland et al. [2020] [Valiente, 2020]). Forthcoming methodological papers will detail our international Consortium's progress throughout 2020‐21, including the conduct of baseline surveys in other countries (e.g. Italy) and follow‐up waves in the UK, Republic of Ireland and Spain, as well as studies of a qualitative and experimental design linked to the UK parent strand.

## 2 METHOD

### 2.1 C19PRC‐UKW1: Fieldwork procedures

#### 2.1.1 Fieldwork organization overview

Fieldwork for the C19PRC was conducted by the survey company Qualtrics. Qualtrics has completed more than 15,000 projects across 2,500 universities worldwide.

#### 2.1.2 C19PRC‐UKW1 sampling design and procedure

The UK adult population aged 18+ years was the target population for C19PRC‐UKW1. Quota sampling methods were employed to achieve a representative sample in terms of age and sex (using 2016 population estimates from Eurostat [2020]) and household income (using 2017 income bands from the Office for National Statistics [2017]).

As an aggregator of panels, Qualtrics provides the online platform to securely house data and leverages partners to connect with respondents. Qualtrics recruits study participants from traditional, actively managed, double‐opt‐in market research panels, that are used for corporate and academic market research only. Qualtrics' partners are members of the European society for opinion and marketing research (ESOMAR), the Council of American Survey Research Organisations (CASRO) and other national organizations.

Potential respondents were alerted to the C19PRC‐UKW1 by Qualtrics in one of two ways: (1) they opted to enter studies they were eligible for by signing up to a panel platform; or (2) they received automatic notification through a partner router that alerted/directed them to studies for that they were eligible (either via email, SMS, and in‐app notifications). Importantly, to avoid self‐selection bias, survey invitations to eligible participants only provide general information and did not include specific details about the contents of the survey. Participants were required to be an adult, able to read and write in English, and a resident of the UK. No other exclusion criteria were applied. Panel members were not obliged to take part; however, members routinely receive an incentive for survey participation based on the length of the survey, their specific panel list profile, and target acquisition difficulty, among other factors. The specific type of reward varies and may include cash, air miles, gift cards, redeemable points, charitable donations, sweepstakes entrance, or vouchers.

Qualtrics proceeded as follows during the six days of fieldwork to fill the quotas (1) respondents in “hard to reach” quota groups (e.g. young adults in the highest income bands) were targeted first; (2) next, the focus shifted to allow the quotas to “fill up” naturally; and (3) finally, a switch back to targeting respondents to fill incomplete quotas ensued. Those who chose to participate followed a link to a secure website and completed all surveys online. The invite link was active for a participant until a quota they would have qualified for was reached but after this time, previously eligible respondents were prevented from taking part.

A power analysis for the C19PRC‐UKW1 sample size was conducted based on an estimate of the population prevalence of the disorder with the lowest rate. The 2014 Adult Psychiatric Morbidity Survey (APMS) in England estimated the rate of PTSD to be 4.4%; this was lower than rates for anxiety and depression (Fear, Bridges, Hatch, Hawkins, & Wessely, 2016). To detect a disorder with a prevalence of 4%, with precision of 1%, and 95% confidence level, a sample size of 1476 was required. However, estimating the prevalence of disorders with a low prevalence (<5%) may result in a small number of “cases” being identified. For instance, a sample size of 1476 and prevalence of 4% will identify approximately 60 cases, and if follow‐up analyses are based on only these cases, then tests may be underpowered. To detect a correlation of 0.30, with alpha = 0.05, and power of 0.80, a sample size of 84 is required. The proportional increase in the sample size required to identify 84 cases results in a sample size of 2100. As a compromise between ensuring adequate sampling to reliably estimate prevalence and ensuring adequate power for sub‐group analysis, a target sample size of 2000 participants was set.

C19PRC‐UKW1 survey data were collected between 23 and 28 March 2020, with two “soft launches”, or pilots, conducted on 19 and 20 March 2020 (*n* = 50 respondents for each launch to check the survey for any errors and/or omissions‐these respondents were excluded from the main sample). Approximately 30 days later, all participants who completed C19PRC‐UKW1 were re‐contacted and invited to participate in C19PRC‐UKW2 during 22 April and 1 May 2020.

#### 2.1.3 Informed consent process

Participants were informed about the purpose of C19PRC Study, that their data would be treated in confidence, that geolocating would be used to determine the area in which they lived (in conjunction with their residential postcode stem) and of their right to terminate participation at any time without giving a reason. Participants were also informed that some topics may be sensitive or distressing. Information about how their data would be stored and analyzed by the research team was also provided. All participants provided informed consent prior to completing the survey and were directed to contact the NHS 111 helpline upon completion if they had any concerns about COVID‐19.

#### 2.1.4 Compliance with General data protection regulation (GDPR)

C19PRC data will be kept confidential in line with GDPR. In accordance with GDPR, contact details were separated from the dataset and personal data is restricted to members of the research team. When the study data is to be deposited with the UK Data Service (see Discussion), location data will be removed and replaced with relevant socioeconomic summary data (e.g. area‐level deprivation and population density data). All other personal data will also be removed.

#### 2.1.5 Quality control

A minimum survey completion time was set at 11 min, 11 s (i.e., half the based on soft‐launch median completion time of 22 min, 22 s). Qualtrics employed checks to identify and remove any participants who (1) completed the survey in less than the minimum completion time (to ensure responses were trustworthy) or (2) were potentially duplicate respondents. Median completion times were 28.91 min for C19PRC‐UKW1 and 36.15 min C19PRC‐UKW2.

### 2.2 Measures

Table 1 provides an overview of the C19PRC survey content (see Supplementary Materials for specific details of all measures). Due consideration was given to the unfolding social, political, and economic events during the design phase of each survey (see Supplementary Table 1 for an example as to how this was conducted in the UK).

**TABLE 1:** Overview of content^a^ of C19PRC study, by survey wave, UK, March–April 2020

Theme	Content	C19PRC Wave	
		1	2
Demographics	Age, gender, ethnicity, country of residence, born in UK, highest level of education, religion, urbanicity, economic activity^a^, key/essential worker status^a^, marital status^a^	X	X^a^ ^only^
Housing characteristics	Number of adults living in household	X	X
	Number of children living in household	X	X
	Ages of children living in household		X
	Housing tenure	X	
	Residential details (type of property; number of bedrooms; length at property; access to open/green space; privacy in residence; broadband availability/suitability)		X
Household finances	Estimated annual gross household income	X	
	Change in monthly household income during pandemic	X	X
	Use of savings/increasing debt during pandemic	X	X
	Lost income due to pandemic	X	
	Made saving due to pandemic	X	
	Concern over household finances being negatively affected due to pandemic	X	X
	Increased purchasing for specific items (e.g. dried food) during pandemic	X	X
Working hours	Number of hours worked weekly pre/post lockdown (self)		X
	Number of adults in household earning income before pandemic		X
	Changes in working hours/employment status of other adult household members		X
Health conditions	Existence of any major underlying health conditions–self	X	X
	Existence of any major underlying health conditions–immediate family member	X	X
	Currently pregnant‐self (partner)	X	X
	Number of weeks pregnant, if applicable	X	X
	Currently pregnant‐immediate family member	X	X
Children in household	Childcare for children in household during lockdown	X	X
	Perception of child's/children's wellbeing during lockdown		X
COVID‐19	Sourcing of information (newspapers, TV, radio, social media, internet, etc.)	X	X
	Level of trust in information source	X	X
	Knowledge of common COVID‐19 symptoms	X	X
	Knowledge of modes of transmission COVID‐19	X	X
	Common beliefs about methods to reduce risk of contracting COVID‐19	X	X
	Avoiding behavior to reduce risk of contracting COVID‐19 (e.g. traveling abroad)	X	
	Engaging in behavior to reduce risk of contracting COVID‐19 (e.g. wearing face mask)	X	X
	Anxiety‐level relating to COVID‐19	X	X
	Perceived risk of serious illness OR death upon contracting COVID‐19: Elderly, children, chronic health conditions, pregnant women	X	X
	Perceived individual risk contracting COVID‐19 over next 6 months	X	X
	Experiences of self‐isolation	X	X
	Eligibility for/experiences of shielding		X
	Experience of being infected with COVID‐19 (self and family member/friend)	X	
	Experience of being tested for COVID‐19 (symptoms/location of testing/diagnosis)		X
	Experience of waiting to be tested for COVID‐19 (self)		X
	Knowing someone close (family member/friend) who has tested positive for COVID‐19		X
	Knowing someone close (family member/friend) who has tested died due to COVID‐19		X
	Competency, opportunity, and motivation to engage in social distancing	X	X
	Competency, opportunity, and motivation to maintain hygiene practices	X	X
	Behavior‐engagement with social distancing	X	X
	Behavior‐engagement with hygiene practices	X	X
	COVID‐19 vaccine acceptability (self)	X	X
	COVID‐19 vaccine acceptability (child)	X	X
	COVID‐19 vaccine acceptability (elderly relative)	X	
	Reasons for accepting COVID‐19 vaccine (self)		X
	Reasons for refusing COVID‐19 vaccine (self)		X
	Information required to accept COVID‐19 vaccine		X
	Willingness to participate in COVID‐19 vaccine trial		X
	General attitudes/beliefs towards vaccines		X
	Conspiracy theories: Conspiracy Mentality Scale (Imhoff & Bruder, 2014)		X
Mental health	Depression: Patient Health Questionnaire‐9 (Kroenke, Spitzer, & Williams, 2001)	X	X
	Anxiety: Generalized Anxiety Disorder Scale‐7 (Spitzer, Kroenke, Williams, & Löwe, 2006)	X	X
	Traumatic stress International Trauma Questionnaire (Cloitre et al., 2018)	X	X
	Paranoia: Persecution and Deservedness Scale (Melo, Corcoran, Shryane, & Bentall, 2009)	X	X
	Somatic symptoms: Patient Health Questionnaire‐15 (Kroenke, Spitzer, & Williams, 2002)	X	X
	Obsessive compulsive behaviors Obsessive Compulsive Inventory‐revized (Foa et al., 2002)		X
	Treatment for mental health difficulties	X	X
	Knowledge about sources of mental health support during pandemic		X
Psychological factors	Personality: Big‐Five Inventory‐10 (Rammstedt & John, 2007)	X	
	Loneliness: Loneliness Scale (Hughes, Waite, Hawkley, & Cacioppo, 2004)	X	X
	Death anxiety: Death Anxiety Inventory (Tomás‐Sábado, Gómez‐Benito, & Limonero, 2005)	X	X
	Religiosity: Monotheist and Atheist Beliefs Scale (Alsuhibani, Shevlin, & Bentall, [2020])	X	
	Locus of control: Locus of Control Scale (Sapp & Harrod, 1993)	X	X
	Self‐esteem: Single‐Item Self‐esteem Scale (Robins, Hendin, & Trzesniewski, 2001)	X	
	Resilience: Brief Resilience Scale ( B. W. Smith et al., 2008)	X	
	Attachment style: Relationships Questionnaire (Bartholomew & Horowitz, 1991)		X
	Intolerance of uncertainty: Intolerance of Uncertainty Scale (Buhr & Dugas, 2002)	X	X
	Blunting/monitoring: Dutch Threatening Medical Situations Inventory (van Zuuren, de Groot, Mulder, & Peter, 1996)		X
	Empathy: Interpersonal Reactivity Index (Davis, 1980)		X
Health‐related behaviors	Alcohol use: AUDIT‐C (Bush, Kivlahan, McDonell, Fihn, & Bradley, 1998)		X
	Smoking		X
	Sleep: Sleep Condition Indicator Scale (Espie et al., 2018)		X
	Daily activities‐ exercise, shopping/socialising/studying		X
Volunteering	Experiences of volunteering during pandemic		X
Family functioning	Family relationship quality pre/post lockdown		X
	Cohabiting partnerships: Division of labor, childcare, domestic violence		X
Socio‐political views/related behaviors	Voting behavior last general election	X	
	Voting behavior European referendum	X	
	Measure of “left‐wing” or “right‐wing” on social and economic issues	X	
	Preference for news source		X
	Satisfaction with how government/institutions handling pandemic		X
	Patriotism/nationalism	X	X
	Identification with humanity: Identification with all humanity scale (McFarland, Webb, & Brown, 2012)	X	X
	Economic individualism and humanitarianism		X
	Social dominance: Social Dominance Scale (Ho et al., 2015)	X	
	Authoritarianism: Very Short Authoritarianism Scale (Bizumic & Duckitt, 2018)	X	
	Attitudes towards migrants	X	
Trust	Other people (general)	X	X
	Institutions	X	X
Belongingness in neighborhood	Connectedness with close neighbor	X	
	Connectedness with wider neighborhood	X	

^a^ Refer to Supplementary Material for detailed information on all study measures.

### 2.3 Ethical approval

Ethical approval for the project was provided by the University of Sheffield (Reference number 033759).

### 2.4 Data analysis plan and weighting procedure

Four sets of analyses are presented to (1) demonstrate the success of the quota sampling methodology at C19PRC‐UKW1; (2) determine the representativeness of the C19PRC‐UKW1 sample to the UK population for a suite of socio‐demographic characteristics not used for quota sampling; (3) describe the socio‐demographic characteristics of the C19PRC‐UKW2 sample, as well as the characteristics of those lost to follow‐up; and (4) estimate the extent to that an a priori selection of socio‐demographic characteristics (age, gender, income, urbanicity, household composition, and having been born/raised in UK) predicted attrition at C19PRC‐UKW2 using a binary logistic regression analysis.

## 3 RESULTS

### 3.1 Quality control checks and representativeness of C19PRC‐UKW1 sample as per quota sampling methods

Given the dual processes used by Qualtrics and partners to recruit respondents to quotas, it was not possible determine the number of survey invites that were distributed to panel members, or indeed the number of panel lists who were alerted to the survey and who did/did not complete the survey (i.e. a response rate). Qualtrics provided metrics for the C19PRC‐UKW1, as follows: (1) having original commenced the survey, 159 respondents did not provide full informed consent and were screened out; 35 respondents who completed the survey from outside the UK or were aged under 18 years (*n* = 6) were also screened out; to ensure responses were trustworthy, 77 participants who completed the survey in less than the minimum completion time were removed, as were 64 potential duplicate respondents. This resulted in a C19PRC‐UKW1 sample of 2025. Table 2 compares the pre‐recruitment quotas to those achieved during the fieldwork period. Sex quotas were obtained to within 1% (slightly more women than men were recruited), age quotas were obtained to within 0.1%–0.6% (fewer respondents aged 25–44 years were recruited), and household income band quotas were obtained to within 0.25%–1% (fewer respondents in the middle income band £25,341–£38,740 were recruited).

**TABLE 2:** Outcome of quota sampling recruitment, COVID‐19 psychological research consortium (C19PRC) study UK Wave 1 (C19PRC‐UKW1), March 2020 (*N* = 2025)

Socio‐demographic characteristics used for quota sampling		Sampling Quota (Target sample *N* = 2000)	Sample Achieved (*N* = 2025)		Percentage difference between sampling quota target and quota obtained
		%	n	%	
Sex^a^	Men	49	972	48.0	−1%
	Women	51	1047	51.8	+0.8%
	Other		6	0.2	NA
Age group (years)^a^	18–24	12	246	12.1	+0.1%
	25–34	19	380	18.8	−0.2%
	35–44	18	353	17.4	−0.6%
	45–54	20	410	20.2	+0.2%
	55–64	17	349	17.2	+0.2%
	65+	14	287	14.2	+0.2%
Gross annual household income^b^	£0–£15,490	20	410	20.2	+0.25%
	£15,491–£25,340	20	410	20.2	+0.25%
	£25,341–£38,740	20	385	19.0	−1.0%
	£38,741–£57,930	20	410	20.2	+0.25%
	£57,931+	20	410	20.2	+0.25%

^a^ Quotas for age and sex were derived from EUROSTAT 2016 population estimates (Eurostat, 2020).

^b^ Quotas for gross household income bands were on 2016 Office for National Statistics data (Office for National Statistics, 2017).

### 3.2 Representativeness of the C19PRC‐UKW1 sample‐UK population estimates.

Reliable estimates for some population estimates (e.g. age, and sex) can be obtained annually from non‐census sources (Office for National Statistics, 2019a). Despite on‐going efforts to develop methods to produce similar reliable mid‐census population estimates for characteristics such as ethnicity (Office for National Statistics, 2019b), the 2011 census remains the most reliable source of population data for many socio‐demographic characteristics (e.g. ethnicity, and country of birth) (Office for National Statistics, 2020), despite the recognition that the population structure has changed since 2011.

Table 3 presents the percentage difference between the proportion of respondents obtained for each socio‐demographic characteristic at C19PRC‐UKW1 compared to the population for each country of the UK. In total, 1951 respondents (95.1% of the sample) provided the stem of their residential postcode. Participants living in England and Wales were combined to facilitate comparison to the 2011 census for England and Wales. The proportion of respondents recruited in Scotland and Northern Ireland was within 0.7% of the 2011 census estimates; fewer participants in England and Wales (5%) were recruited when compared to population estimates.

**TABLE 3:** Comparison of representativeness of the COVID‐19 psychological research consortium (C19PRC) study UK Wave 1 (C19PRC‐UKW1) sample to UK adult population for key socio‐demographic characteristics, by country, March 2020 (*N* = 1951)

		Sample (%)			Comparison to UK adult population (+/−% difference between survey sample and population)		
		England/Wales	Scotland	Northern Ireland	England/Wales (*N* = 42,645,389)	Scotland (*N* = 4,109,000)	Northern Ireland (*N* = 1,329,919)
Country of residence^a^		83.7%	7.8%	2.3%	88.7% (−5.0%)	8.5% (+0.7%)	2.8% (−0.5%)
Ethnicity^b^	White British/Irish	85.8%	89.5%	90.9%	83.0% (+2.8%)	93.1% (−3.6%)	98.2% (−0.5%)
	White other	5.5%	6.6%	6.8%	4.7% (−0.8%)	3.3% (+3.3%)	
	Indian	2.2%	0%	0%	2.5% (−0.3%)	0.6% (−0.6%)	‐
	Pakistani	1.1%	2.0%	0%	1.6% (−0.5%)	0.8% (+1.2%)	‐
	Chinese	1.0%	0.7%	0%	0.8% (+0.2%)	0.7% (0.0%)	‐
	Black/African/Afro‐Caribbean	2.1%	0.7%	0%	2.9% (−0.8%)	0.5% (−0.2%)	‐
	Arab	0.2%	0%	0%	0.4% (−0.2%)	0.2% (+0.2%)	‐
	Bangladeshi	0.3%	0%	0%	0.6% (−0.3%)	0.1% (−0.1%)	‐
	Other Asian	0.4%	0%	2.3%	1.4% (−1.0%)	0.4% (−0.4%)	‐
	Other	1.5%	0.7%	0%	2.1% (−0.6%)	0.3% (+0.4%)	1.8% (+1.8%)
Economic activity^c^	Full‐time (including self‐employed)	49.1%	46.7%	43.2%	43.5% (+5.6%)	43.3% (+3.4%)	42.5% (+0.7%)
	Part‐time (including self‐employed)	14.3%	16.4%	22.7%	16.8% (−2.5%)	15.8% (+0.6%)	15.1% (+7.6%)
	Unemployed (looking for work)	5.0%	5.3%	11.4%	4.1% (+0.9%)	4.3% (+1.0%)	7.4% (+4.0%)
	Unemployed (not looking for work)	6.7%	5.3%	6.8%	6.3% (−0.4%)	5.2% (+0.1%)	5.0% (+1.8%)
	Retired	17.1%	17.8%	6.8%	22.8% (−5.7%)	23.7% (−5.9%)	12.9% (−6.1%)
	Student	4.6%	2.6%	4.5%	2.4% (+2.2%)	2.6% (0.0%)	9.8% (−5.3%)
	Not looking for work (e.g. due to disability)	3.2%	5.9%	4.5%	4.1% (0.9%)	5.1% (+0.8%)	7.3% (−2.8%)
Born in the UK^d^	Yes	90.5%	92.8%	90.9%	84.5% (+6.0%)	92.8% (0.0%)	92.6% (−1.7%)
	No	9.5%	7.2%	9.1%	15.5% (−6.0%)	7.2% (0.0%)	7.4% (+1.7%)
Household composition^e^	Lone adult only household	22.1%	27.6%	18.2%	25.6% (−3.5%)	33.1% (−5.5%)	‐
	Other	77.9%	72.4%	81.8%	74.4% (+3.5%)	66.9% (+5.5%)	‐

^a^ 3.7% of the survey sample did not provide data relating to postcode stem and therefore country of origin could not be established.

^b^ Source: 2011 Census population estimates for adults aged 18+ years for England/Wales and Scotland; adults aged and Northern Ireland. Tai & Sun, 2011 Census remains the most reliable source of population data for many socio‐demographic characteristics (e.g. ethnicity, country of birth), despite the recognition that the population structure has changed since 2011.

^c^ Source: 2011 Census population estimates for adults aged 20+ years for England/Wales and Scotland; Northern Ireland age 16–74 years (no other breakdown of age publicly available).

^d^ Source: 2011 Census population estimates for adults aged 25+ years for England/Wales and Scotland; adults aged 18+ years for Northern Ireland.

^e^ Source: 2011 Census population estimates for adults aged 25+ years for England/Wales and Scotland; Northern Ireland provides publicly available data on household composition for the household reference person only (*N* = 703,275), not for all adults aged 18+ years, and therefore a comparison to survey for household composition is not feasible.

The ethnic profile of respondents was diverse and closely mirrored that of the UK population. Specifically, for England/Wales, the proportion of White British/Irish was higher than expected (2.8%) and the proportion of White Other was lower (0.8%); in Scotland and Northern Ireland, the proportion of respondents in the two White categories combined was within 0.5% of population estimates, although in Scotland the proportion of White British/Irish was lower than expected (3.6%) but White Other was higher (3.3%), suggesting some variation in the self‐categorization as White among Scottish respondents in the survey. In England/Wales and Scotland, non‐White ethnic groups were well‐represented and the proportions were achieved to within 1% of population estimates; in Northern Ireland, population estimates are only provided at a higher level for minority ethnic groups (1.8% of population), but the ethnic profile of the sample suggested these respondents were largely of Asian ethnicity.

The economic activity profile of the C19PRC‐UKW1 sample was comparable to population estimates (aged 20+ years, see Table 2 footnote); the most notable differences between the sample and population occurred in relation to full‐time employment (i.e., higher proportions of respondents from England/Wales and Scotland were in full‐time employment [49.3% and 46.7%, respectively] compared to the 2011 census [43.5% and 43.3%, respectively], part‐time employment in England/Wales [14.3% achieved, 16.8% expected]), and retirement (i.e., lower proportions of respondents living in England/Wales 5.7%] and Scotland [5.9%] reported that they were retired than reported in the 2011 census). More students (2.2%) were recruited in England/Wales than would have been expected, but all other economic activity categories were obtained to within 1% of population estimates. For Northern Ireland, there was generally good representation of all economic activity groups, albeit there were higher proportions of adults who were unemployed or employed part‐time, but fewer retirees or students than expected.

A higher proportion of respondents (90.5%) than expected (84.5%) in England/Wales were born in the UK, although the sample proportion obtained for Scotland was identical to the 2011 Scottish Census. In Northern Ireland, a lower proportion of respondents reported having been born in the UK compared to the population estimates (90.9% compared to 92.6%, respectively). Finally, with respect to household composition, the proportion of respondents living in “lone adult only” households in both England/Wales and Scotland was lower than expected (similar comparison for Northern Ireland were not feasible‐see Table 3).

### 3.3 Follow‐up of C19PRC‐UKW1 sample at C19PRC‐UKW2

A total of 1508 respondents initiated the C19PRC‐UKW2 interview (overall re‐contact rate of 74.5%) but 102 respondents did not complete the survey in full. Of these, the majority were female (52.9%), aged 65 years or older (29.4%), and earned £25,341–£38,740 per year (25.4%). Analysis of survey data revealed that the majority of these respondents (71.6%) appeared to click on the survey link but did not complete the informed consent section; the remaining respondents initiated the survey, but exited out between 36 s and 29.2 h (median exit time 15 min, 30 s). The final C19PRC‐UKW2 study sample of 1406 respondents represented an actual retention rate of 69.4%.

### 3.4 Socio‐demographic characteristics of C19PRC‐UKW1/C19PRC‐UKW2 samples (*n* = 2025; *n* = 1406)

Table 4 compares the socio‐demographic characteristics of the C19PRC‐UKW1 and C19PRC‐UKW2 sample. Results from the binary logistic regression analysis (model not present) revealed that all variables selected a priori were significant bivariate predictors of attrition and the overall multivariate model was significant (χ^2^ (14) = 317.54, *p* < 0.001); the C19PRC‐UKW2 sample had more males, older people, those in higher income brackets, those of White ethnicity, people living outside cities, those living in lone adult‐only households, and individuals born/raised in the UK. Subsequently, an inverse probability weighting procedure (Seaman & White, 2013) was conducted to ensure that the C19PRC‐UKW2 sample was representative of the C19PRC‐UKW1 sample.

**TABLE 4:** Sociodemographic characteristics of respondents, COVID‐19 psychological research consortium (C19PRC) study UK Wave 1 (C19PRC‐UKW1), March 2020 and Wave 2 (C19PRC‐UKW2), April 2020

Sociodemographic characteristics		C19PRC‐UKW1 (*N* = 2025)		C19PRC‐UKW2 (*N* = 1406–69.4% retention rate)	
		*N*	%	*N*	%
Sex	Male	972	48.0	732	52.1
	Female	1047	51.8	67	47.7
	Other	6	0.2	4	0.2
Age group (years)	18–24	246	12.1	77	5.5
	25–34	380	18.8	217	15.4
	35–44	353	17.4	246	17.5
	45–54	410	20.2	311	22.1
	55–64	349	17.2	304	21.6
	65+	287	14.2	251	17.9
Gross household income	£0–£15,490	410	20.2	279	19.8
	£15,491–£25,340	410	20.2	252	17.9
	£25,341–£38,740	385	19.0	259	18.4
	£38,741‐£57,930	410	20.2	311	22.1
	£57,931+	410	20.2	305	21.7
Ethnicity	White British/Irish	1732	85.5	1239	88.1
	White non‐British/Irish	116	5.7	68	4.8
	Indian	41	2.0	26	1.8
	Pakistani	27	1.3	13	0.9
	Chinese	19	0.9	15	1.1
	Afro‐Caribbean	13	0.6	5	0.4
	African	27	1.3	10	0.7
	Arab	3	0.1	3	0.2
	Bangladeshi	6	0.3	4	0.3
	Other Asian	11	0.5	3	0.2
	Other	30	1.5	20	1.4
Urbanicity	City	498	24.6	307	21.8
	Suburb	572	28.2	433	30.8
	Town	620	30.6	418	29.7
	Rural area	335	16.5	248	17.6
Number of adults in household	1	454	22.4	351	25.0
	2	1132	55.9	794	56.5
	3	270	13.3	166	11.8
	4	130	6.4	77	5.5
	5+	39	1.9	18	1.2
Number of children (under 18 years in household)	0	1433	70.8	1045	74.3
	1	293	14.5	184	13.1
	2	238	11.8	143	10.2
	3	44	2.2	7	0.5
	4+	17	0.8	5	0.9
Born in UK	Yes	1834	90.6	1239	92.0
	No	191	9.4	113	8.0
Raised in the UK	Yes	1872	92.4	1311	93.2
	No	153	7.6	95	6.8

## 4 DISCUSSION

Despite the urgency for scientific evidence to help inform the global response to the rapidly evolving COVID‐19 pandemic, it has been emphasized recently that now, more than ever, research studies must be of high quality (Nieto, Navas, & Vázquez, 2020) and that this can only be achieved through the use of focused questions, the employment of robust methodologies and the securement of necessary ethical approval(s) from relevant institutions (Hipp, Bünning, Munnes, & Sauermann, 2020; Townsend, Nielsen, Allister, & Cassidy, 2020; World Health Organisation, 2020c). We believe that, through the design and initiation of a robust, representative, longitudinal, multi‐country study, during the earliest phases of the SARS‐CoV‐2 pandemic, the C19PRC study surpasses these research quality standards.

Several strengths of the C19PRC ensure that this study's data will be well placed to make a significant contribution to the knowledge base surrounding the COVID‐19 pandemic, over the short and long term, including: (1) the multidisciplinary, multi‐country composition of the C19PRC affords a valuable opportunity to study the psychosocial impact of COVID‐19 from an ecological perspective that considers the influences of social, political, media, economic, and demographic factors on the psychological health and wellbeing of the population, and how these vary across countries; (2) the recruitment of a large nationally representative adult sample in each country ensures that C19PRC study findings have strong generalizability; (3) the broad and deep coverage of a wide range of important individual‐level psychosocial risk/protective factors and outcomes warranting long‐term investigation during a pandemic; (4) the ability to enhance the quality and explanatory power of individual‐level survey data through the prioritization of important geo‐spatial data collection, that permits linkage to “macro‐level” data (e.g. country‐level population characteristics; COVID‐19 related statistics, etc.); (5) the longitudinal design of the study, the first two waves of that were conducted within one‐month of each other (producing a robust follow‐up rate of 69.4%), that enabled the C19PRC to analyze, and report on study data in the most timely fashion, that, in turn, provides maximum opportunity for the study findings to aid and inform important clinical and policy‐related decision making; and (6) securing permission to re‐contact members from the survey “spine” for additional “sub‐projects”. Such projects, to include methodologies such as qualitative interviewing, experimental designs, and experience sampling methodologies, as well as other “spin‐off” studies involving adolescents and young people (University of Sheffield, 2020), will provide a unique opportunity to apply a mixed‐methods approach to uncover important aspects of the pandemic, as they unfold over time, in greater detail.

As is common with all studies, the C19PRC study is not without limitations and chief among these is the use of quota sampling to recruit the non‐probability based sample via the internet. This opt‐in mode of recruitment employed by Qualtrics, albeit being a cost‐effective method for gaining fast access to a large and diverse sample (and the only feasible method of recruitment during the pandemic), inevitably meant that it was not possible to generate a response rate for the baseline survey due to the lack of a known denominator or sampling frame. While more research is required to fully investigate the strengths and weaknesses associated with internet‐based panel surveying (Bergeson, Gray, Ehrmantraut, Laibson, & Hays, 2013), it has been suggested that the composition of non‐probability internet‐based survey panels differs from that of the underlying population (Hays, Liu, & Kapteyn, 2015). For example, recent evidence suggests that individuals who participate in online surveys during the pandemic and who tend to be lazy (as measured by the Big Five Personality Inventory‐10) have a higher participation probability and a lower probability to comply with behavioral measures and practices intended to reduce the risk of the spread of SARS‐CoV‐2 (Schaurer & Weiß, 2020); therefore, when generalizing the results of internet‐based panel survey findings to the general population, there may be a risk of underestimating the proportion of the population that comply with such measures. More generally, the American Association for Public Opinion Research (APPOR) asserts that when non‐probability sampling (as opposed to probability sampling) methods are used, there is a higher burden of responsibility on investigators to describe the methods used to draw the sample and collect the data, so that users can make an informed decision about the usefulness of the resulting survey estimates (Baker et al., 2013). We support the APPOR's position that it is useful to think of different non‐probability sampling approaches as falling on a continuum of expected accuracy of the survey estimates; at one end are uncontrolled convenience samples that produce risky survey estimates by assuming that respondents are a random sample of the population, whereas at the other end, there are surveys that recruit respondents based on criteria related to the survey subject matter and then the survey results are adjusted using variables that are correlated with the key study outcome variables (Baker et al., 2013). The design of C19PRC ensures that it falls towards the latter end of the continuum. Despite the demonstrated representativeness of the C19PRC‐UKW1 sample, the Consortium are cognizant that the sample composition recruited at baseline and retained at first follow‐up may make specific sub‐group analyses (e.g. between‐country; potentially vulnerable groups of the population, such as frontline/key workers and/or ethnic minority respondents) difficult. Our plans to recruit additional survey members into the study in subsequent waves of the UK parent strand, with a specific focus on over‐sampling respondents in Wales, Scotland, and Northern Ireland, and among underrepresented sub‐groups of the population, where possible, shall facilitate more robust comparisons of this nature for the core study mental health outcomes. Details of this methodological approach will be described in forthcoming methodological papers from the C19PRC study.

The C19PRC study data will facilitate and stimulate interdisciplinary research on important public health questions such as: (1) What role does the public's knowledge, attitudes, behaviors, and practices have in determining health outcomes during the COVID‐19 pandemic? (2) What level of trust does the public have in public/political institutions and how is this associated with compliance with COVID‐19 related health/protective/preventative behaviors or practices? (3) What is the psychological impact and sequalae of COVID‐19 and its associated socio‐economic effects in the UK? (4) Who is most at risk of psychological distress during COVID‐19? (5) What does resilience look like in the context of COVID‐19 and what factors contribute to it? and (6) How do the public feel about future vaccination for COVID‐19? Findings from the C19PRC study on the topics of mental health disorders and COVID‐19 related health behaviors have already been accepted for publication (Gibson Miller et al., 2020; Shevlin et al., 2020). The C19PRC has recently received funding from the UKRI/ESRC COVID‐19 rapid response call, supporting on‐going data collection for the parent strand of the study into mid‐2021. Finally, our consortium is committed to depositing our survey data with the UK Data Archive Service and to actively and widely encouraging re‐use of the data so that maximum benefit can be achieved from this robust data resource.

## CONFLICT OF INTEREST STATEMENT

All authors declare no conflict of interest.

## Supporting information

Supplementary Material 1

Supplementary Material 2
