# Supplementary material for: Monitoring the psychological, social, and economic impact of the COVID‐19 pandemic in the population: Context, design and conduct of the longitudinal COVID‐19 psychological research consortium (C19PRC) study
Source: Int J Methods Psychiatr Res. 2020 Nov 9;30(1):e1861. doi: 10.1002/mpr.1861 (PMC7992290; doi:10.1002/mpr.1861)
Supplement: Supplementary file 1 — Supplementary Material 1 [file MPR-30-e1861-s001.docx]

Monitoring the psychological, social, and economic impact of the COVID-19 pandemic in the population: Context, design and conduct of the longitudinal COVID-19 Psychological Research Consortium (C19PRC) Study

**Supplementary Material**

Orla McBride^1*^, Jamie Murphy^1^, Mark Shevlin^1^, Jilly Gibson-Miller^2^, Todd K. Hartman^2^, Philip Hyland^3^, Liat Levita^2^, Liam Mason^5^, Anton P. Martinez^2^, Ryan McKay^5^, Thomas VA Stocks^2^, Kate M. Bennett^6^, Frédérique Vallières^7^, Thanos Karatzias^8^, Carmen Valiente^9^, Carmelo Vazquez^9^, & Richard P. Bentall^2, 6^

^1^ Ulster University, Northern Ireland

^2^ University of Sheffield, England

^3^ Maynooth University, Republic of Ireland

^4^University College London, England

^5^ Royal Holloway, University of London, England

^6^ University of Liverpool, England

^7^Trinity College Dublin, Republic of Ireland

^8^Napier University, Scotland

^9^Complutense University of Madrid

Short title: C19PRC Study protocol

**Corresponding author:** Dr Orla McBride, School of Psychology, Ulster University, Cromore Road, Coleraine, BT52 1SA, Northern Ireland. Email: [o.mcbride@ulster.ac.uk](mailto:o.mcbride@ulster.ac.uk); Tel: 0044(0)2870123987.

**2. Methods**

**2.2 Measures**

**2.2.1 Socio-demographic characteristics.** In addition to data relating to gender, age, and gross annual household income (which were used for quota sampling), respondents provided data on their: ethnicity (White British/Irish; White non-British/Irish; Indian; Pakistani; Chinese; Afro-Caribbean; African; Arab; Bangladeshi; other Asian; or other-specify); religion (Christian; Muslim; Jewish; Buddhist; Sikh; Atheist; Agnostic; or other-specify); urbanicity of residence (city; suburb; town; or rural area); highest level of education (no qualifications; O-level/GCSE or similar; A-level or similar; diploma; undergraduate degree; postgraduate degree; technical qualification; or Other-specify); legal marital or same-sex status (never married and never registered same-sex civil partnership (single); separated, but still legally married; divorced; widowed; married; in a registered same-sex civil partnership; separated but still legally in a same-sex civil partnership; or formally in a same-sex civil partnership which is now legally dissolved; surviving partner from a same-sex civil partnership); born in UK (yes/no); raised in UK (before age of 16 years) (yes/no); and country of residence (England, Wales, Scotland, or Northern Ireland).

**2.2.2 Economic activity***.* CP19PRC-UKW1: Respondents were asked to indicate whether they were: employed full-time (including self-employed); employed part-time (including self-employed); unemployed (looking for work); unemployed (not looking for work); not in employment (due to disability); retired; or student. C19PRC-UKW2: Additional information was sought in relation to the respondent’s current employment status (employed full-time; employed part-time (regular hours); retired; zero hour contract; other flexible work practice; unemployed (not due to COVID-19); or unemployed (due to COVID-19). Respondents who indicated that they were employed were also asked whether they were: an employee, self-employed, a business owner or other (specify). Respondents were also asked to consider a definition of a ‘key worker’ (i.e. people whose jobs are vital to public health and safety during the coronavirus lockdown) and to determine whether their occupation was covered by any of the following categories (UK Cabinet Office/Department of Education, 2020): (1) Health and social care worker (e.g. all NHS staff including administrative and cleaning staff, care home workers); (2) Education and child care (e.g. nursery care workers and teachers); (3) Food and other necessary goods (e.g. staff involved in production, processing, distribution, sale and delivery of goods); (4) Key public services (e.g. postal workers, those required to run the justice system, religious staff, those responsible for managing the deceased and journalists providing public service broadcasting); (5) Local and national government (e.g. staff in administrative roles essential to the effective delivery of the COVID-19 response or delivering essential public services including payment of benefits); (6) Utility workers (e.g. staff needed to keep oil, gas, electricity, water and sewerage operations running, staff in the civil nuclear, chemical and telecom communications sectors); (7) Public safety and national security (e.g. police and support staff, Ministry of Defence civilian staff and armed forces personnel, fire and rescue staff, and workers responsible for border security, prisons, and probation); or (8) Transport (e.g. staff keeping air, water, road and rail passenger and freight transport modes operating).

**2.2.3 Housing characteristics.**

*2.2.3.1 Household composition.* C19PRC-UKW1: Respondents provided information as to whether they lived alone or, if they lived with other people, how many adults and/or children aged younger than 18 years, lived with them. Respondents were also asked about housing tenure (own outright; own with a mortgage; shared ownership; renting; living rent free; or other). C19PRC-UKW2: Data was collected about the age of each child (from 0-17 years) living in the household, where applicable, starting with the youngest child.

*2.2.3.2 Physical properties of place of residence*. C19PRC-UKW2***:*** Respondents were asked to indicate: (1) the type of property in which they live (a flat, house or bungalow); (2) how many bedrooms are in the property (ranging from 0 bedrooms, a single-room dwelling, studio or flat to 5 or more); and (3) how long they have lived at the property (under 1 year, 1-2 years, 3-5 years, or over 5 years). Respondents were also asked whether their residence had a private garden, a shared garden, or a balcony (Yes/No response). Respondents also reported on the amount of private space they have in their home and the extent to which they agree with the following statements (scored on a 7-point Likert scale ranging from strongly disagree to strongly agree, not applicable option included): (1) If someone else is watching TV or playing music in the living area, there is somewhere you can go that is suitable and quiet; (2) The size and layout of your home does not allow you enough privacy; (3) Your children have a room with enough space in which they can play alone; (4) Your children can do their homework in private at a desk or table; (5) You can work in private at a desk or table; and (6) Your broadband coverage is suitable for your work or social networking needs.

**2.2.4 Household finances.**

*2.2.4.1 Income changes during the pandemic.* C19PRC-UKW1: Respondents were asked to report their experiences of changes in income during the pandemic, including: (1) a loss of income due to not being able to work as much or because business contracts had been cancelled or delayed; and (2) any financial savings made due to changes in leisure activities (e.g. not eating out in restaurants; attending the cinema or sports events). Response scales for both items were ‘my household has/has not lost/saved income because of the COVID-19 pandemic’ (with the additional option of ‘unsure whether my household has/has not lost/saved income because of the COVID-19 pandemic). C19PRC-UKW1/C19PRC-UKW2*:* Respondents were asked to estimate the percentage change in their monthly household income, compared to the average monthly income before the pandemic, on a visual slider scale centred at 0 and ranged from 100% (decrease) on the left-hand side to 100% (increase) on the right-hand side. Respondents were also asked whether they were using savings to help their household survive during the crisis and whether they were going further into debt because of the crisis (Yes/No response option). Finally, respondents were asked to report, on balance, how much they had worried about the way their household finances had been affected by the pandemic so far, with response options ranging from 1 ‘not at all worried’ to 10 ‘extremely worried’.

*2.2.4.2 Purchasing behaviours.* C19PRC-UKW1/C19PRC-UKW2: A series of questions were developed to measure respondents’ purchasing behaviours during the early phases of the pandemic. Respondents were asked to report the extent to which they increased their purchasing of the following items in the weeks before the survey: (1) Tinned food; (2) Water; (3) Sanitary products (e.g. hand sanitiser); (4) Toilet roll; (5) Dried foods (e.g. pasta. rice); (6) Bread; (7) Pharmacy products (e.g. painkillers, cold/flu products); (8) Batteries; (9) Fuel (heating or car fuel); and (10) alcohol. Responses were recorded on a five-point Likert scale ranging from (1) ‘not at all’ to 5 ‘very considerably’.

*2.2.4.3 Working hours.* C19PRC-UKW2: Respondents were asked about general changes to their working hours during the lockdown and whether they: (1) had continued to work normal hours; (2) were working more hours; (3) were working reduced hours; (4) been placed on the Government ‘furlough scheme’; or (5) stopped working for the time being. This question was followed-up by asking respondents the number of hours they worked per week prior to the lockdown and during the lockdown, with responses for each time period scored on a 6-point scale: 0 ‘0-10 hours’, 1 ’11-20 hours’, 2 ’21-30 hours’, 4 ’31-40 hours’, 5 ’41-50 hours’, or 6 ‘50+ hours’. Respondents who lived in a household with other adults were asked to report how many of these adults were earning an income before the pandemic (response scale 0 to 4 or more) and whether, during the pandemic, any of these adults: (1) had continued to work normal hours; (2) were working more hours; (3) were working reduced hours; (4) been placed on the Government ‘furlough scheme’; or (5) stopped working for the time being (Yes/No response option).

**2.2.5 Health conditions***.* C19PRC-UKW1/C19PRC-UKW2: Following guidance from the UK National Health Service (NHS) (NHS, 2020b), the UK government directed that living with a major underlying health conditions is a risk factor for experiencing more severe ill-health and even death upon contracting COVID-19. Participants were asked whether they and members of their immediate family were living with any major underlying health conditions (e.g. lung conditions, heart disease, kidney disease, liver disease, conditions affecting the brain and nerves, diabetes, problems with your spleen, a weakened immune system) (Yes/No response). Respondents were asked whether they or their partner were pregnant at the time of the survey and, if so, how many weeks? Respondents were also asked whether any members of their immediate family were pregnant at the time of the survey.

**2.2.6 Children in household.**

*2.2.6.1 Childcare.* C19PRC-UKW2: Where applicable, information was requested in relation to who is taking care of children in the household during the lockdown while childcare facilities and schools are closed/unavailable: (1) mother or main female carer; (2) father or main male carer; (3) both parents equally; (4) grandmother; (5) grandfather; (6) an immediate family member; (7) an extended family member; (8) a family friend; (9) availing of key worker childcare support; (10) mixture of several options; or (11) other (specify).

*2.2.6.2 Wellbeing.* C19PRC-UKW2: Respondents who reported that dependent children were living in the household were asked the extent to which they agreed with a series of statements relating to their child/children’s general wellbeing (scored on a 5-point Likert scale from strongly disagree to strongly agree), as follows: (1) On the whole, my child/children are coping well with the current situation; (2) I am worried that I am not able to provide good home schooling for my school age child/children; (3) I am enjoying being able to spend more time with my child/children; (4) My child/children seem more anxious; (5) There is now less conflict in the home.

**2.2.7 COVID-19.** As this study was devised in mid-March 2020 at the beginning of the pandemic when COVID-19 was a new virus, no existing measure was available to assess the general population’s knowledge, attitudes, and behaviours (KAB) of the virus. In order to assess COVID-19 related KAB, measures developed for use in studies of other global pandemics, for example the 2003 SARS outbreak, the 2009 H1N1 flu pandemic and the 2013-16 Ebola virus pandemic, were consulted and assessed for suitability and adaptation, where possible. Reliable and trusted web sources in the UK (e.g. Public Health England, the National Health Service; NHS) and internationally (e.g. the Centre for Disease Control, the WHO) were also consulted for current, evidence-based knowledge and information relating to the clinical presentation and transmission of COVID-19. Details of the newly devised questions/measures are described below.

*2.2.7.1 Sourcing information about COVID-19 and level of trust in sources*. C19PRC-UKW1/C19PRC-UKW2: Respondents were asked about (1) how much information about COVID-19 that they had obtained from a variety of sources, including newspapers, television, radio, internet websites, social media, their doctor, other health professionals, government agencies, and family or friends?; and (2) how much trust they had in the information they got from each source? Responses were scored on a 4-point Likert scale ranging from 1 ‘None/Not at all’ to 4 ‘A lot’.

*2.2.7.2 Confidence in UK response to COVID-19.* C19PRC-UKW2: A series of questions were developed to ascertain respondents’ level of satisfaction (with response options ranging from 0 ‘Not at all satisfied’ to 100 ‘Extremely satisfied’) with how specific institutions and figures were responding to the COVID-19 crisis: (1) the UK government; (2) Prime Minister, Boris Johnson; (3) Matt Hancock, Secretary of State for Health and Social Care; (4) Rishi Sinak, Chancellor of the Exchequer; (5) Public Health Officials; (6) the National Health Service (NHS); and (7) the Bank of England.

*2.2.7.3 Knowledge of COVID-19 symptoms.* C19PRC-UKW1/C19PRC-UKW2*: Respondents were asked to indicate, based on current knowledge, what they believed to be the most commonly reported symptoms of COVID-19 from a list of 12 symptoms (yes/unsure/no response): fever; vomiting; tiredness; muscle pains/aches; coughing; rash; diarrhoea; severe headache; loss of taste or smell*****; breathing difficulties/shortness of breath; bleeding (internal or external); sore throat; nasal congestion. According to CDC/WHO, the three most common symptoms of COVID-19 (in March-April 2020) were a cough, fever, shortness of breath; ‘yes’ responses to all three symptoms would indicate accurate knowledge of COVID-19 symptoms (Centre for Disease Control and Prevention, 2020a; World Health Organisation, 2020a). Symptoms such as bleeding, vomiting, a rash, and diarrhoea were common with other global pandemics (e.g. Ebola), whereas symptoms such as sore throat, headaches and muscle aches are more commonly associated with cold or influenza (Australian Government Department of Health, 2020).

*2.2.7.4 Transmission of COVID-19*. C19PRC-UKW1/C19PRC-UKW2: Respondents were asked a series of statements relating to possible pathways of transmission of COVID-19 (“Based on current knowledge, how do you think COVID-19 spreads? Can the virus be spread by…?”) and were required to indicate whether they believed (yes/no) these to be correct. Eight modes of transmission included: people touching each other; people coughing or sneezing; food contamination; insects (e.g. flies); breathing the air outside; breathing the air in confined spaces; contact with pets; and touching surfaces. According to the Centre for Disease Control and Prevention (2020c), extant knowledge indicated that the COVID-19 virus transmitted person-to-person via two main modes of transmission: close physical human contact and through respiratory droplets produced when an infected person sneezes or coughs. Accurate knowledge of COVID-19 transmission in this survey, therefore, is indicated by respondents correctly identifying ‘no’ to modes of transmission involving food contamination, insects, breathing air outside, and contact with pets.

*2.2.7.5 Attitudes relating to risk of contracting COVID-19*. C19PRC-UKW1/C19PRC-UKW2: To assess level of engagement with, and retention of, information provided in ongoing national and international public health campaigns (Centre for Disease Control and Prevention, 2020b; NHS, 2020a; World Health Organisation, 2020b), respondents were asked the extent to which they agreed with six attitudinal statements relating to general risk of contracting COVID-19 (scored on a 5-point Likert scale ranging from 1 ‘completely disagree’ to ‘5 completely agree’): (1) antibiotics are effective in preventing and treating COVID-19; (2) washing your hands with soap and water, or using alcohol-based hand-rub regularly, may help reduce risk of infection; (3) healthy people without symptoms should wear a face mask; (4) regularly rinsing your nose with saline will help reduce the risk of COVID-19; (5) cold weather helps to kill the COVID-19 virus; and (6) maintaining at least 2 metres (6 feet) distance between yourself and another person ('social distancing'), may help reduce your risk of infection. Agreement (or strong agreement) with statements 2 and 6 was deemed to be indicative of engagement with current public health messaging.

*2.2.7.6 Behaviour change to reduce individual risk of contracting COVID-19*. C19PRC-UKW1: Respondents were asked whether they had, more generally, changed their behaviours following best practice advice from the earliest messages in public health campaigns: “To protect yourself from COVID-19, to what degree have you changed your plans and behaviour to avoid the following?”: (1) Travelling to infected areas (e.g. China, Italy); (2) Travelling via airplane; (3) Travelling in taxis; (4) Travelling on public transport (e.g. trains, underground, buses); (5) Close contact greetings with other people (e.g., shaking hands, hugging); (6) Eating in restaurants; (7) Attending large gatherings of people (e.g. cinema, theatre, concerts); (8) Touching your eyes or mouth; (9) Being close to people who are ill; (10) Going to school, college or work; and (11) Taking children to school. Responses ranged from 1 ‘Not at all’ to 4 ‘Avoided completely’. C19PRC-UKW1/C19PRC-UKW2: Respondents were asked to report on whether and how they have changed behaviours relating to their personal care and health recently to reduce their personal risk of being infected by COVID-19 during the pandemic. Statements were posed as follows: “To reduce your risk of being infected by COVID-19 have you recently…”: (1) Worn a face mask; (2) Washed your hands with soap and water more often; (3) Used hand sanitising gel if soap and water were not available; (4) Used disinfectants to wash surfaces in your home more frequently; (5) Covered your nose and mouth with a tissue or sleeve when coughing or sneezing; (6) Taken a herbal supplement; and (7) Ensured you have enough sleep. Response categories were no, occasionally, or whenever possible. Responses ranged from 1 ‘Not at all’ to 4 ‘Avoided completely’.

*2.2.7.7 Anxiety relating to COVID-19.* C19PRC-UKW1/ C19PRC-UKW2: Respondents’ degree of specific anxiety about the COVID-19 pandemic was assessed using a single visual slider scale, ranging from 0 ‘not at all anxious’ on the left-hand side to 100 ‘extremely anxious’ on the right-hand side.

*2.2.7.8 Perceived risk of contracting COVID-19.* C19PRC-UKW1/ C19PRC-UKW2: Respondents estimated on a visual slider (ranging from 0% on the left-hand side to 100% on the right-hand side) their perceived percentage risk of contracting COVID-19 within the next month, within 2-3 months, and within 4-6 months of the survey. They were also asked to estimate the perceived risk of different vulnerable groups – the elderly, children, pregnant women, and those with any major underlying chronic health conditions – experienced serious illness and (separately) death following a diagnosis of COVID-19 – using the same ‘percentage risk’ visual slider.

*2.2.7.9 Experiences of self-isolation.* C19PRC-UKW2***:*** Respondents were presented with a definition of self-isolation (‘*self-isolation means that you have COVID-19 symptoms, or if someone you live with has symptoms, you must not leave your home for between 7-14 days’*) and were asked whether they were currently self-isolating (Yes/No response) and (separately) whether they had self-isolated in the past (Yes/No response).

*2.2.7.10 Experiences of shielding*. C19PRC-UKW2: Respondents were also presented with a definition of shielding (‘*shielding is a measure to protect people with serious underlying health conditions by minimising all interaction with others (health conditions such as e.g. lung conditions, heart disease, kidney disease, liver disease, conditions affecting the brain and nerves, diabetes, problems with your spleen, a weakened immune system’*) and were asked to report whether they were eligible for shielding (Yes/No response). Respondents who indicated they were eligible to shield were asked (1) whether they had been contacted by the Government by text or post to inform them that they must shield (Yes/No response); and (2) whether they had been able to shield (Yes/No response). Respondents who were eligible for shielding, but who had not been contacted by the Government, were asked whether they had tried to contact the NHS or Government to inform them of their eligibility for shielding (Yes/No response). Those who were eligible for shielding, but were unable to do so, were provided with a text box to provide a response explaining why they were unable to shield. All respondents who reported they were eligible for shielding were asked whether (1) they able to get deliveries of essential items including (i) groceries and other household items from a short or supermarket and (ii) medication and other things needed to stay healthy from a doctor or pharmacist; and (2) whether family and friends had helped them of get essential items of (i) and (ii) (Yes/No response to all questions).

*2.2.7.11 Testing for COVID-19 – self.* C19PRC-UKW2: Respondents were asked whether they had been tested for COVID-19 (Yes/No response). For those who had been tested, further information was sought in relation to (1) whether they had experienced the main symptoms of the virus (e.g. fever, persistent cough) when they received the test (Yes/No response); (2) where they received the test – at home, at a GP’s practice, in a hospital, at a designated COVID-19 test centre, or other (specify); (3) the outcome of the test i.e. the test was positive (respondent received a diagnosis of COVID-19) or negative (respondent was not diagnosed with COVID-19); (4) whether they were admitted to hospital (Yes/No response); and (5) how unwell they felt when experiencing COVID-19 (response scale was a visual slider ranging from 0 ‘Not at all unwell’ to 100 ‘Extremely unwell’. Respondents who had not been tested for COVID-19 were asked whether they were currently waiting on a test (Yes/No response) and, for those who were waiting for a test, they were asked to report how long they had been waiting (response scale ranging from 1 ‘1-2 days’ to 5 ’More than 14 days).

*2.2.7.12 Testing for COVID-19 – other.* C19PRC-UKW2: Respondents were asked (Yes/No response) whether anyone else from their household had been diagnosed with COVID-19 (confirmed by a test) and whether anyone from their extended family or network of friends had been diagnosed with COVID-19 (confirmed by a test).

*2.2.7.13 COVID-19-related deaths.* C19PRC-UKW2: Respondents were asked whether anyone close to them had died because of COVID-19, with response options including ‘Yes’, ‘No’ and ‘Unsure – not certain death was caused by COVID-19’.

*2.2.7.14 Competency, opportunity, and motivation to engage in health behaviours to reduce risk of COVID-19 transmission**.* C19PRC-UKW1/C19PRC-UKW2: Seventeen questions based on the COM-B (Capability, Opportunity, Motivation-Behaviour) model of behaviour change (Michie, Van Stralen, & West, 2011) assessed respondents’ ability to engage in two key public health-protective behaviours recommended to help prevent COVID-19 and reduce or slow the spread of the disease, namely, *maintaining hygienic practices* and *social distancing*. Questions focused on respondents’ perception of the extent to which they experienced sufficient motivation, capability, and opportunity to enact the recommended behaviours. Items were adapted from a preliminary version of the COM-B self-evaluation questionnaire (COM-B-Qv1) (Michie, Atkins, & West, 2014). In relation to each of the two health-protective behaviours, participants were asked to indicate the extent to which seventeen statements were true for them during the COVID-19 pandemic on a 5-point Likert scale ranging from 1 ‘strongly agree’ to 5 ‘strongly disagree’. Definitions of each behaviour were given within the questions. *Maintaining hygienic practices* was defined as ‘for example hand washing frequently, cleansing surfaces, avoid touching eyes and mouth, using tissues’ and *social distancing* was defined as ‘for example staying in your home most of the time, exercising outside once a day, not meeting up with friends and relatives, maintaining a 2-metre distance from people, working from home)’. Capability was measured by three items: “I knew about why it was important and had a clear idea about how the virus was transmitted”, “I knew about how and when to do it” and “I was able to overcome the physical and/or mental barriers that might have stopped me from doing it”. Opportunity was measured by six items, split into physical opportunity: “I had the necessary time to do it”, “It was easy for me to do it”, “People were doing it around me”, “I had reminders that prompted me” and social opportunity: “I had support from others” and “I felt like doing it was normal and expected”. Motivation was measured by eight items, split into five items measuring reflective motivation: “I intended to do it”, “I felt that I wanted to do it”, “I believe that it was a good thing to do”, “I developed a specific plan for doing it”, “I developed a habit of it in my everyday routine” and three items measuring automatic motivation: “It made me feel anxious”, “It made me feel disgusted” and “I felt like I could control my emotional reactions so I could do it”.

*2.2.7.15 Health behaviour engagement -* S*ocial distancing and Maintaining hygiene practices.* C19PRC-UKW2: Respondents were asked, ‘In the past week, to reduce your risk of being infected by or passing on the coronavirus to others, on how many days of the week have you: (1) left the house for food, health reasons, or work; (2) exercised outside once a day (either alone or with members of your household); (3) exercised outside more than once a day; (4) met up with friends or extended family (outside of your home); (5) gathered in a group of more than two people in a park or other public space; (6) driven to a national park or other green space to exercise; (7) left the house to provide assistance to a vulnerable or elderly person; (8) worked from home; (9) washed your hands as soon as you returned home after being outside; (10) stayed at least 2 metres (6ft) away from others when in public; (11) reminded your children about hygienic practices or social distancing rules; (12) engaged in close contact greetings with people outside of your family (e.g. shaking hands, hugging); (13) experienced disapproval from others when out of the house, whether or not it was for a good reason; (14) found it difficult to find a place or space to exercise whilst observing the social distancing rules; (15) been instructed to go home, leave an area or dispersed by the police; and (16) been taken home, arrested or fined by the police for breaking the social isolation rules? Responses were recorded on a 5-point scale ranging from 0 ‘Not at all’ to 4 ‘Every day’.

*2.2.7.16 COVID-19 vaccine – part 1.* At the time this survey was designed, no vaccine was available for COVID-19. In anticipation of a vaccine being developed in the future, a series of questions were developed to ascertain respondents’ views about a potential vaccine, adapting items from existing measures designed to access vaccine acceptability for other diseases (see below). C19PRC-UKW1/C19PRC-UKW2*. Respondents were asked “*If a new vaccine were to be developed that could prevent COVID-19, would you accept it for: (1) yourself********; (2) your child/children*****; (3) elderly relatives”, with three response options (Yes, No, Maybe).

*2.2.7.17 COVID-19 vaccine - part 2.* C19PRC-UKW2*.* Respondents who indicated they would accept a vaccine were asked to indicate, on a slider scale of between 0 ‘Not at all’ and 100 ‘A lot’, the extent to which the following statements reflected why they had responded positively: (1) I said yes because I generally feel positive about vaccinations; (2) I said yes because I trust how vaccines are developed, tested, and administered to the public; and (3) I said yes because I believe that the consequences of contracting COVID-19 outweigh the potential side effects of a vaccine.

Adults who responded with ‘no or maybe’ to the acceptability of a potential COVID-19 vaccine were asked a series of additional questions, developed following consultation with a range of sources and measures (Horne, Powell, Hummel, & Holyoak, 2015; Martin & Petrie, 2017; Shapiro, Holding, Perez, Amsel, & Rosberger, 2016; Yaqub, Castle-Clarke, Sevdalis, & Chataway, 2014): ‘*We would like to know a little more about your response to the COVID-19 vaccine question. Which of any of the following influenced your response*…”: (1) I have an underlying medical condition that would prevent me from getting a COVID-19 vaccine; (2) I would not want to risk vaccine injury; (3) I am terrified of needles; (4) the potential side effects of the vaccine could be worse than the symptoms of COVID-19; (5) I am not part of a group considered ‘at-risk’ for serious COVID-19 health outcomes; (6) a COVID-19 vaccine would make a lot of money for pharmaceutical companies, but not do much for regular people; (7) I will not allow the government or any doctor to tell me what to do with my body; and (8) being exposed to the COVID-19 virus naturally would be safer for the immune system than being exposed through vaccination. Responses were recorded on a visual slider scale ranging between 0 ‘Not at all’ and 100 ‘A lot’ and a free-text box was also provided for respondents to indicate any other information which contributed to their ‘no or maybe’ response.

Adults who responded with ‘no or maybe’ to the acceptability of a potential COVID-19 vaccine were also presented with a series of statements in an attempt to gauge what information they might require to convince them to accept a vaccine, as follows: (1) clear information on the effectiveness of the vaccine; (2) if the vaccine had undergone rigorous scientific trials; (3) if other countries had already begun to use the vaccine; (4) clear information about the potential side effects or risks of the vaccine; (5) knowing that the vaccine is available at no financial cost; (6) a recommendation from my religious or spiritual leader (i.e. Iman, Priest, Rabbi); and (7) an endorsement from the political party I belong to. Responses were recorded on a visual slider scale ranging between 0 ‘not at all’ and 100 ‘a lot’.

All respondents were also asked to indicate they extent to which they agreed with a series of statements about vaccinations more generally (scored on 5-point Likert scale from ‘strongly disagree’ to ‘strongly agree’): (1) I generally feel positive about vaccinations; (2) I am opposed to vaccinations under any circumstances generally, no matter what; (3) I trust how vaccines are developed, tested, and administered to the public; (4) people are deceived about vaccine safety and efficacy; (5) some vaccines cause serious health problems in otherwise healthy people; (6) authorities promote vaccines for financial gain, not for people's health; (7) vaccinating healthy people helps protect others by stopping the spread of disease; (8) modern medicine offers a poor understanding of how to achieve health and wellbeing; (9) being exposed to a virus naturally is safer for someone’s immune system rather than being exposed through vaccination; and (9) the risks of childhood vaccines for measles, mumps and rubella outweigh the benefits. Finally, respondents were asked whether they would volunteer to participate in a trial for a COVID-19 vaccine (Yes/No response).

*2.2.7.18 COVID-19 conspiracy theories.* C19PRC-UKW2***:*** A set of statements were designed to assess adults’ knowledge of and engagement with prominent theories about the origins of the COVID-19 in circulation at the time of the survey (April 2020). Respondents were asked the extent to which they agreed (using a visual slider scale ranging from 0% ‘Do not believe at all’ to 100% ‘Completely believe’) with the following statements: (1) COVID-19 was developed in a lab in Wuhan, China; (2) COVID-19 originated from a meat market in Wuhan, China; (3) 5G mobile networks are responsible for the current global pandemic; (4) Coronavirus is actually no more dangerous than the common flu; and (5) High doses of Vitamin C can cure Covid-19.

**2.2.8 Mental health.** Experiences of mental health difficulties are core outcomes for the C19PRC Study. A key objective of the study was to administer a range of brief, standardised questionnaires to screen for the presence of common mental disorder, which would be repeated across all survey waves. Details are included below.

*2.2.8.1* *Patient Health Questionnaire-9 (PHQ-9*) (Kroenke, Spitzer, & Williams, 2001). C19PRC-UKW1/C19PRC-UKW2: Depression was assessed with the PHQ-9, a nine-item measure which corresponds to the DSM-IV Diagnostic Criterion A symptoms for major depressive disorder (American Psychiatric Association, 2000). Participants were asked how often, over the last two weeks, they had been bothered by each of the depressive symptoms. Response options were “not at all”, “several days”, “more than half the days”, and “nearly every day”, scored as 0, 1, 2 and 3, respectively. PHQ-9 scores range from 0 to 27, with scores of ≥5, ≥10, ≥15, representing mild, moderate and severe levels of depression severity (Kroenke et al., 2001). A threshold of ≥10 was used in this study. Psychometric properties of the PHQ-9 are well documented (see Kroenke, Spitzer, Williams, and Löwe (2010) for an overview).

*2.2.8.2 Generalized Anxiety Disorder Scale (GAD-7)* (Spitzer, Kroenke, Williams, & Löwe, 2006). C19PRC-UKW1/C19PRC-UKW2: Experiences of generalized anxiety were assessed using the GAD-7. Respondents were asked to report, on a 4-point Likert scale ranging from 1 (not at all) to 4 (nearly every day), how often in the past 7 days they were bothered by seven anxiety symptoms (e.g. trouble relaxing, becoming easily annoyed or irritable). The GAD-7 was originally validated in a primary care sample and a cut-off score of 10 had a sensitivity value of 0.89 and a specificity value of 0.82 for identifying generalised anxiety disorder (Spitzer et al., 2006), and a threshold of 10 was used in this study. The GAD-7 has demonstrated good reliability and construct validity, as evidenced by strong associations with other established measures of anxiety as well as diagnoses of GAD and its associations with depression, self-esteem, life satisfaction, and resilience (Löwe et al., 2008).

*2.2.8.3 International Trauma Questionnaire (ITQ)* (Cloitre et al., 2018). C19PRC-UKW1/C19PRC-UKW2: Post-traumatic stress disorder was assessed using the ITQ, a self-report measure of ICD-11 PTSD based on a total of six symptoms across the three symptom clusters of Re-experiencing, Avoidance, and Sense of Threat; each symptom cluster is comprised of 2 symptoms. Participants were asked to complete the ITQ as follows: “…in relation to your experience of the COVID-19 pandemic, please read each item carefully, then select one of the answers to indicate how much you have been bothered by that problem in the past month”. The PTSD symptoms are accompanied by three items measuring functional impairment caused by these symptoms. All items are answered on a 5-point Likert scale, ranging from 0 (Not at all) to 3 (Extremely) with possible PTSD scores ranging from 0 to 24. A score of ≥ 2 (Moderately) is considered ‘endorsement’ of that symptom. A PTSD diagnosis requires traumatic exposure, and at least one symptom to be endorsed from each PTSD symptom cluster (Re-experiencing, Avoidance, and Sense of Threat), and endorsement of at least one indicator of functional impairment. The psychometric properties of the ITQ scores have been demonstrated in multiple general population (Ben‐Ezra et al., 2018; Cloitre et al., 2019) and clinical and high-risk samples (Hyland et al., 2017; Karatzias et al., 2016; Vallières et al., 2018) samples.

*2.2.8.4 Patient Health Questionnaire-15 (PHQ-15)* (Kroenke, Spitzer, & Williams, 2002). C19PRC-UKW1/C19PRC-UKW2: The PHQ-15 is a brief, self-administered questionnaire which assesses for the presence and severity of the most prevalent DSM-IV somatization disorder somatic symptoms (American Psychiatric Association, 2000). Respondents rated the severity of symptoms, such as stomach pain, headaches, dizziness, they experienced over the last seven days as 0 (‘not bothered at all’), 1 (‘bothered a little’) or 2 (‘bothered a lot’). PHQ-15 score ranges from 0 to 30 and scores of ≥5, ≥10, ≥15 represent mild, moderate and severe levels of somatization (Kroenke et al., 2002). The reliability and validity of the PHQ-15 are high in clinical and occupational health care settings (de Vroege, Hoedeman, Nuyen, Sijtsma, & van der Feltz-Cornelis, 2012; Kroenke et al., 2002; Kroenke et al., 2010).

*2.2.8.5 Persecution and Deservedness Scale* (PaDS) (Melo, Corcoran, Shryane, & Bentall, 2009). C19PRC-UKW1/C19PRC-UKW2*:* Paranoia was assessed with five items taken from the persecution subscale of the persecution and deservedness scale (PaDS), a measure designed for use with both clinical and population samples and which has been validated against both questionnaire and clinical measures of paranoia (Elahi, Algorta, Varese, McIntyre, & Bentall, 2017; Melo et al., 2009). Participants rated their agreement on a 5-point scale with statements such as “I’m often suspicious of other people’s intentions towards me” and “You should only trust yourself.” Response options ranged from 1 = strongly disagree to 5 = strongly agree. Scale reliability for the five items was very good (α = 0.84) in a previous epidemiological study of UK citizens (McIntyre, Wickham, Barr, & Bentall, 2018).

*2.2.8.6 Obsessive Compulsive Inventory-Revised (OCI-R)* (Foa et al., 2002). C19PRC-UKW2: The OCI-R is an 18-item scale which is used to assess six types of obsessive-compulsive behaviours: washing (e.g. *I sometimes have to wash or clean myself simply because I feel contaminated*); checking/doubting (e.g. *I repeatedly check doors, windows, drawers, etc*.); obsessing (e.g. *I find it difficult to control my own thoughts*); mental neutralising (e.g. *I feel compelled to count while I am doing things*); ordering (e.g. *I get upset if others change the way I have arranged things*); and hoarding (e.g. *I have saved up so many things that they get in the way*). All items are scored on a 5-point Likert scale ranging from 1 ‘Not at all bothered/distressed’ to 5 ‘Extremely distressed/bothered’. The OCI-R has good to excellent internal consistency, test–retest reliability, and convergent validity (Foa et al., 2002).

*2.2.8.7 Treatment seeking behaviour for mental health difficulties*. C19PRC-UKW1/C19PRC-UKW2: All respondents were asked about their history of mental health difficulties, with the following response categories: I never received treatment for mental health problems; I have received treatment for mental health problems in the past; I am currently receiving treatment for mental health problems; I am currently receiving treatment for mental health problems, but it has been cancelled temporarily due to the lockdown; and I’d prefer not to answer. C19PRC-UKW2: Adults currently in receipt of treatment for mental health problems were asked how they were accessing their treatment during the lockdown (response options: by phone, online (e.g. using Skype/Zoom) or other (specify)).

*2.2.8.8 Receipt of advice/awareness of public health messages in relation to mental health care during lockdown.* C19PRC-UKW2: The C19PRC Study project partner, Public Health England, requested that the inclusion of a question to assess whether respondents had *“received or seen any specific advice about how to maintain your mental health during the coronavirus crisis?”* (Yes/No response). Adults indicating they had received/seen specific advice were asked to indicate the sources of that advice from a range of options including: (1) a newspaper or magazine; (2) online (other than a newspaper or magazine); (3) a television programme; (4) Public Health England, Public Health Scotland, Public Health Wales, or HSC Public Health Agency (Northern Ireland); or (5) a health professional.

**2.2.9 Psychological factors.**

*2.2.9.1 Big-Five Inventory (BFI-10)* (Rammstedt & John, 2007). C19PRC-UKW1: The five personality traits of openness to experience, conscientiousness, extroversion, agreeableness and neuroticism were assessed using the BFI-10, which contains items two items per personality construct such as ‘I see myself as someone who is reserved’, ‘ I see myself as someone who tends to be lazy’, and ‘I see myself as someone who has few artistic tendencies’. Rammstedt and John (2007) reported good reliability and validity for the 10-item scale.

*2.2.9.2 Loneliness Scale* (Hughes, Waite, Hawkley, & Cacioppo, 2004). C19PRC-UKW1/C19PRC-UKW2: Social connectedness was measured using the three-item Loneliness Scale, which was specifically designed for use in large-scaled population surveys (Hughes et al., 2004). Respondents were asked how often they felt: (1) that they lacked companionship; (2) left out; and (3) isolated from others. Responses were scored on a 3-point scale (hardly ever, sometimes, or often).

*2.2.9.3 Single-Item* *Self-esteem Scale* (SISES) (Robins, Hendin, & Trzesniewski, 2001). C19PRC-UKW1: Respondents’ reported the extent to which they agreed with a single statement (‘I have high self-esteem’) on a 7-point Likert scale ranging from 1 ‘not very true of me’ to 7 ‘very true of me’. The SISES has been shown to have good convergent validity against other self-esteem measures (Robins et al., 2001).

*2.2.9.4 Brief Resilience Scale (BRS)* (Smith et al., 2008). C19PRC-UKW1: Respondents’ level of resilience was assessed using the 6-item BRS, which included the items such as: ‘I tend to bounce back quickly after hard times’; ‘ I have a hard time making it through stressful events’; and ‘ I tend to take a long time to get over set-backs in my life’. Items were scored on a 5-point Likert scale ranging from 1 ‘strongly disagree’ to 5 ‘strongly agree’, with items 2,4 and 6 reverse coded. The BRS has demonstrated construct, convergent, and discriminant validity in the general population (Kyriazos et al., 2018; Rodríguez-Rey, Alonso-Tapia, & Hernansaiz-Garrido, 2016).

*2.2.9.5 Locus of control (LoC) scale* (Sapp & Harrod, 1993). C19PRC-UKW1/C19PRC-UKW2: The short 9-item version of Levenson’s LoC scale (Levenson, 1973), which measures internal LoC (items such as ‘My life is determined by my own actions’) and external LoC, which has two components - change (items such as ‘To a great extent, my life is controlled by accidental happenings’) and powerful others (items such as ‘Getting what I want requires pleasing those people above me’). The internal, chance, and powerful others subscales were each measured by three questions using a 7-point Likert scale ranging from 1’ strongly disagree’ to 7 ‘strongly agree’.

*2.2.9.6* *Death Anxiety Inventory (DAI)* (Tomás-Sábado, Gómez-Benito, & Limonero, 2005). C19PRC-UKW1/C19PRC-UKW2: Respondents’ attitudes towards death were assessed using the 17-item DAI, which measures four death-related anxiety factors (labelled as death acceptance, externally generated death anxiety, death finality, and thoughts about death) with items such as ‘I get upset when I am in a cemetery’, ‘The sight of a corpse deeply shocks me’, ‘I find it difficult to accept the idea that it all finishes with death’ and ‘I find it really difficult to accept that I have to die’. Responses were scored on a 5-point Likert scale ranging from 1 ‘totally disagree’ to 5 ‘totally agree’ (Tomás-Sábado et al., 2005).

*2.2.9.7 Facial detection of trust* (Martinez, Agostini, Alsuhibani, & Bentall, in submission). C19PRC-UKW1: This task used stimuli obtained from the trustworthiness dataset of the Princeton Social Perception Lab database (Oosterhof and Todorov (2008). This dataset contains computer-generated faces created using FaceGen 3.1 and includes identities manipulated on different traits as rated by a normative sample (attractiveness, competence, dominance, extroversion, likeability, threat, and trustworthiness). From the data set, 10 bald Caucasian male computer-generated faces (6 prior rated as trustworthy and 6 prior rated as untrustworthy) were randomly selected by using the website [www.Random.org](http://www.Random.org). Participants were presented with each face and were asked: “*How much would you trust this person*”. Answers were binary (1 = *“I would not trust this person*” to 7 = *“I would trust this person”*) .

*2.2.9.8* *Cognitive Reflection Task of Analytical Reasoning (CRT).* C19PRC-UKW1: CRT is a measure of analytical reasoning (Frederick, 2005). Respondents’ level of ability was assessed using an adapted version which included two additional items as well as the three in the original scale CRT. Respondents were asked to solve the following five problems, each of which is designed to stimulate intuitively appealing but incorrect responses: (1) A bat and a ball cost £1.10 in total. The bat costs £1.00 more than the ball. How much does the ball cost? (2) If it takes 5 machines 5 minutes to make 5 widgets, how long would it take 100 machines to make 100 widgets? (3) In a lake, there is a patch of lily pads. Every day, the patch doubles in size. If it takes 48 days for the patch to cover the entire lake, how long would it take for the patch to cover half of the lake? (4) If you’re running a race and you pass the person in second place, what place are you in? (5) A farmer had 15 sheep and all but 8 died. How many are left? Each problem hints at an incorrect answer and analytic reasoning (or ‘slow thinking’; (Kahneman, 2012) reflects correct responses obtained by ignoring or discounting the hinted answer. The response format was multiple choice with three foil answers (including the hinted incorrect answer) as recommended by Sirota and Juanchich (2018).

*2.2.9.9 Relationships Questionnaire (RQ)* (Bartholomew & Horowitz, 1991). C19PRC-UKW2: The RQ is a four-item scale designed to measure adult attachment styles. Respondents are presented with four statements capturing general relationship styles (A-D) and asked to indicate which letter best describes them, and then to indicate on a 7-point Likert scale, ranging from 1 ‘Strongly disagree’ to 7 ‘Strongly agree’, how well or poorly each description corresponds to your general relationship style: Style A (*It is easy for me to become emotionally close to others. I am comfortable depending on them and having them depend on me. I don’t worry about being alone or having others not accept me*); Style B (I am uncomfortable getting close to others. I want emotionally close relationships, but I find it difficult to trust others completely, or to depend on them. I worry that I will be hurt if I allow myself to become too close to others); Style C (*I want to be completely emotionally intimate with others, but I often find that others are reluctant to get as close as I would like. I am uncomfortable being without close relationships, but I sometimes worry that others don’t value me as much as I value them*); and Style D (*I am comfortable without close emotional relationships. It is very important to me to feel independent and self-sufficient, and I prefer not to depend on others or have others depend on me*).

*2.2.9.10 Intolerance of uncertainty* (Buhr & Dugas, 2002). C19PRC-UKW1/C19PRC-UKW2*:* Respondents’ intolerance of uncertainty, which is thought to play a key role in the aetiology and maintenance of worry, was assessed using the 12-item Intolerance of Uncertainty Scale (IUS) (Buhr & Dugas, 2002). The IUS has a good construct validity (Birrell, Meares, Wilkinson, & Freeston, 2011), with two factors ‘*unexpected events are negative and should be avoided*’ measured by items such as ‘I always want to know what the future has in store for me’, and ‘*uncertainty leads to the inability to act*’ measured by items such as ‘the smallest doubt can stop me from acting’. All 12 items are scored on a 5-point Likert scale ranging from 1 ‘not at all characteristics of me’ to 5 ‘entirely characteristic of me’. The IUS has excellent internal consistency, good test–retest reliability over a five-week period, and convergent and divergent validity when assessed with symptom measures of worry, depression, and anxiety (Buhr & Dugas, 2002).

*2.2.9.11 Dutch Threatening Medical Situations Inventory (TMSI)* (van Zuuren, de Groot, Mulder, & Peter, 1996). C19PRC-UKW2: TMSI was developed to measure cognitive confrontation (‘monitoring’) and cognitive avoidance (‘blunting’) within the domain of medical threat. It consists of four scenarios of threatening medical situations followed by monitoring and blunting alternatives. The monitoring scale of the TMSI pertains to three different item-contents: a) looking for information within the threatening situation b) going deeply into the situation by reading about it, and c) getting information about the situation from other doctors, patients, or organisations. The blunting scale refers to two item-contents: a) seeking distraction away from the threatening situation and b) having an optimistic viewpoint. The shortened version of the TMSI was used (Ong et al., 1999), which consists of two out of the four hypothetical situations, pertaining to 'vague suspicious headache complaints' and 'choosing for uncertain heart surgery'. Respondents were asked to read a scenario (“*Imagine you suffer from headaches and dizziness for some period of time already. You visit your doctor. He or she tells you things don’t look too well and refers you to a specialist for a rather trying medical examination*”) and report the extent to which they agree with four statements (scored on a 5-point scale ranging from ‘not at all applicable to me’ to ‘strongly applicable to me’: (1) I plan to ask the specialist as many questions as possible; (2) I plan to start reading about headaches and dizziness; (3) For the time being I try not to think of unpleasant outcomes; and (4) I am not going to worry: such an examination is not as bad as suffering from headaches all the time. Next, respondents were asked to consider a different scenario (“*Now imagine you are experiencing heart problems. You see a specialist. He or she tells you that you will need surgery, but the success is uncertain*.”) and report the extent to which they agree with four statement using the same response scale: (1) I plan to get as much information about heart surgery as possible; (2) I intend to contact patients who have the same problem, to get information; (3) I’ll assume I will benefit from the operation; and (4) I am thinking: it will all turn out alright.

*2.2.9.12 Interpersonal Reactivity Index (IRI)* (Davis, 1980, 1983). C19PRC-UKW2: The IRI is by far the most widely used instrument to assess empathy (Pulos, Elison, & Lennon, 2004). The 28-item self-report measure consists of four, 7-item subscales (empathetic concern, personal distress, fantasy, and perspective-taking), which are measured on a 6-point Likert scale ranging from 1 ‘strongly disagree’ to 6 ‘strongly agree’. Two subscales of the IRI - perspective-taking (cognitive empathy) and empathic concern (affective empathy) - were administered to respondents. Examples of cognitive empathy included “*Before criticizing somebody, I try to imagine how I would feel if I were in their place*” and “*I believe that there are two sides to every question and try to look at them both*”, whereas examples of affective empathy items included “*I often have concerned feelings for people less fortunate than me*” and “*When I see someone being treated unfairly, I don't feel much pity for them*”. Both subscales have very good reliability (perspective-taking, α=0.79; emphatic concern, α=0.80) (Pulos et al., 2004).

**2.2.10 Health-related behaviours.**

*2.2.10.1 Sleep Condition Indicator (SCI)* (Espie et al., 2018). C19PRC-UKW2: The SCI is an eight-item scale, comprising (1) two quantitative items on sleep continuity (getting to sleep; remaining asleep); (2) two qualitative items on sleep satisfaction/dissatisfaction (sleep quality; troubled sleep or not); (3) two quantitative items on severity of sleep problem (nights per week; duration of sleep problem) and (4) two qualitative items on attributed daytime consequences of poor sleep on personal functioning (impact on mood, energy or relationships) and daytime performance (impact on concentration, productivity, or ability to stay awake). Total scores on the SCI range from 0-32, which higher scores indicating better sleep. In a study using data from 200,000 adults, Espie et al. (2018) demonstrated that the SCI has acceptable internal consistency (α=0.733) and test-re-test reliability (*r*=0.84).

*2.2.10.2 Alcohol Use.* C19PRC-UKW2*:* Questions were drawn from the 2014 Adult Psychiatric Morbidity Survey (McManus, Bebbington, Jenkins, & Brugha, 2016) to ascertain whether respondents currently drink alcohol nowadays, including drinks they brew or make at home (Yes/No response). Current drinkers were asked to complete an adapted version of the 3-item AUDIT-C (Bush, Kivlahan, McDonell, Fihn, & Bradley, 1998), which has good specificity and sensitivity for detecting alcohol dependence and at-risk drinking in the general population (Dawson, Grant, Stinson, & Zhou, 2005). The AUDIT-C questions were asked with reference to use prior to the lockdown (23^rd^ March 2020) as follows: (1) how often did you have a drink containing alcohol (response categories 1 ‘never’ to 5 ‘4 or more times a week’; (2) how many drinks containing alcohol did you have on a typical day when you were drinking (response categories 1 ‘1-2 drinks’ to 5 ’10 or more’; and (3) how often did you have six or more alcohol drinks on one occasion (response categories 1 ‘never’ to 5 ‘almost daily’.

*2.2.10.2.1 Drinking Motivation Questionnaire* (DMQ) (Cooper, Russell, Skinner, & Windle, 1992). C19PRC-UKW2: The DMQ is a 15-item scale that assesses the relative frequency of drinking for three conceptually and empirically distinct motive dimensions (enhancement, social and coping) in the past week. Each dimension consists of five items such as: how often do you drink to forget your worries (e.g., a coping motive), to have fun (e.g. enhancement motive), or to celebrate (e.g. a social motive)? Responses were rated on a 3-point frequency scale ranging from 1 ‘never/almost never’ to 3 ‘almost always’. The DMQ has good internal consistency and construct validity (Gilson et al., 2013). Minor revisions to the wording of some items were made to reflect drinking motivations during the lockdown period.

*2.2.10.2.2 Context of drinking* *during lockdown*. C19PRC-UKW2: Respondents were asked the extent to which they drank alcohol in the past week in the following settings: (1) on your own, in your house/garden; (2) with someone else in your own house/garden; (3) with family/friends online (e.g. WhatsApp, group Zoom/Skype); (4) in public (i.e. outside your house/garden). Responses were rated on a 4-point frequency scale ranging from 1 ‘Never’ to 4 ‘always’.

*2.2.10.3 Tobacco Use.* C19PRC-UKW2: Questions were drawn from the 2014 Adult Psychiatric Morbidity Survey (McManus et al., 2016) to ascertain whether respondents ever had smoked a cigarette (Yes/No response) and whether they smoke at all nowadays (Yes/No response). Current smokers were asked to report the number of cigarettes they smoked on average per day pre-lockdown and since the lockdown started. Response categories: 1 ‘1 cigarette’, 2 ’2-5 cigarettes’, 3 ‘6-10 cigarettes’, 4 ’11-20’cigarettes’ or 5 ‘20+ cigarettes’.

*2.2.10.4 Daily activities.* C19PRC-UKW2*:* Respondents were provided with a list of 12 general daily activities the public are permitted to engage in during the lockdown (e.g. went shopping, exercise inside the home; exercised outside the home; spent time on a hobby; used social media to socialise, etc.) and asked to indicate, in the last full day prior to completing the activity; (1) how many times they had engaged in each activity (response scale 1 ‘Not at all’ to 4 ‘3 or more times’); and (2) for what duration (response scale 1 ‘1-30 minutes’, 2 ‘1 hours’ or 3 ‘More than one hour’. *Interaction with other people*. Respondents were also asked to report “*In total, how many people have you talked to face-to-face today (not via telephone, Skype or any other electronic method). Include anyone who you do not share your home with – e.g. someone you met at the checkout of a supermarket, friend seen while walking in the park, someone who comes to deliver groceries*)”, on scale ranging from 0 ‘0 people’ to 5 ‘5 or more people’.

**2.2.11 Volunteering.** C19PRC-UKW2: Respondents were to consider the following statement “*During the pandemic, people have been volunteering to help in different ways. Some people have been trying to boost morale in their communities by decorating their houses or clapping to show support for the NHS, for example. Others have been joining community networks or local action groups to support people in their community, and many people have been volunteering through charities or with the NHS directly to support responses to the pandemic*”, and indicate the extent to which they had engaged in: (1) boosting community morale during the pandemic; (2) volunteering for a charity action/community groups to help people cope with the pandemic; (3) volunteering with a charity to help with their response to the pandemic; and (4) volunteering through the NHS to help with their response to the pandemic. Responses were scored on a 6-point scale ranging from 1 ‘I am actively involved in this activity’ to 6 ‘I would not consider engaging in this activity’.

**2.2.12 Family functioning.**

*2.2.12.1 Brief Family Relationship Scale (BFRS)* (Fok, Allen, Henry, & Team, 2014). C19PRC-UKW2: The BFRS is adapted from the 27-item Relationship dimension of the Family Environment Scale (FES) (Moos, 1994), consisting of cohesion, expressiveness, and conflict subscales (9 items each, response options range from 0 ‘Strongly agree’ to 3 ‘Strongly disagree’). These subscales measure support, expression of opinions, and angry conflict within a family. For this survey wave, the BFRS was adapted to measure family relationships with reference to two time periods (before and during the lockdown) and the response format was adapted to a 3-point scale ranging from 1’Not at all’ to 3 ‘A lot’.

*2.2.12.2 Cohabiting relationship changes since lockdown*. C19PRC-UKW2: Respondents were asked their experiences of cohabiting during the lockdown via the following statements (1) Compared with my partner, I have carried most of the responsibility of caring for our child/ren; (2) Compared with my partner, I have been mostly responsible for housework and cooking; (3) Compared with my partner, I have carried most of the responsibility of caring for elderly or vulnerable relatives/neighbours/friends; (4) I have felt unsafe in my home; and (5) I have experienced violence or abuse from my partner. Responses scored on 3-point scale from 1 ‘Not at all’ to 3 ‘More than usual’ (not applicable option).

**2.2.13 Social and political attitudes and behaviours.**

*2.2.13.1* *Very Short Authoritarianism Scale (VSA)* (Bizumic & Duckitt, 2018). C19PRC-UKW1: Under the Dual-Process Motivational Model (Duckitt, 2001, 2009), right-wing authoritarianism (RWA) and social dominance orientation (SDO) are conceptualised as value-attitude-belief dimensions which emerge from two different motivational schemas: threat-control (RWA) and competition-dominance (SDO). Both are robust predictors of a range of right-wing political beliefs, including prejudice. Past research also demonstrates that RWA can interact with the perception of threat to produce support for anti-democratic policies (Cohrs, Maes, Moschner, & Kielmann, 2007; Kossowska et al., 2011). The six-item VSA was used to assess respondents’ levels of RWA, and includes items such as: ‘It’s great that many young people today are prepared to defy authority’; ‘What our country needs most is discipline, with everyone following our leaders in unity’; and ‘Our society does NOT need tougher government and stricter laws’. All items were scored on a 5-point Likert scale ranging from 1 ‘strongly disagree’ to 5 ‘strongly agree’, with three items reverse coded.

*2.2.13.2* *Social Dominance Scale* (SDO_7_) (Ho et al., 2015). C19PRC-UKW1: Respondents’ levels of social dominance orientation were assessed using the eight-item SDO_7._ Respondents were asked the extent to which they opposed/favoured statements such as : ‘An ideal society requires some groups to be on top and others to be on the bottom’; ‘Some groups of people are simply inferior to other groups’; and ‘We should do what we can to equalize conditions for different groups’. Response were scored using a 5-point Likert scale ranging from 1 ‘Strongly oppose’ to 5 ‘Strongly Favour’. Ho et al. (2015) demonstrated the SDO_7_ had good criterion and construct validity.

*2.2.13.3 Conspiracy mentality scale (CMS)* (Imhoff & Bruder, 2014). C19PRC-UKW1: Conspiracy mentality is a generalized political attitude, distinct from established generalized political attitudes like right-wing authoritarianism (RWA) and social dominance orientation (SDO) (Imhoff & Bruder, 2014). Respondents completed five items of the CMS (scored on a 5-point scale from 1 ‘Certainly not 0%’ to 11 ‘Certainly 100%’), including: ‘I think that many very important things happen in the world, which the public is never informed about’; ‘I think that politicians usually do not tell us the true motives for their decisions’; and ‘ I think that there are secret organizations that greatly influence political decisions’.

*2.2.13.4 Patriotism/Nationalism*. C19PRC-UKW1/C19PRC-UKW2: Items to measure patriotism and nationalism were adapted from Davidov (2011). Patriotism was assessed by pride in Britain’s democracy, its National Health Service (NHS) and its fair and equal treatment of all groups in society. Nationalism was assessed by two items: ‘The world would be a better place if people from other countries were more like the British’ and ‘Generally speaking, Britain is a better country than most other countries’. Responses were scored on 5-point Likert scales from 1 ’strongly disagree’ to 5 ’strongly agree’.

*2.2.13.5 Identification with all humanity scale (IWAH)* (McFarland, Webb, & Brown, 2012). C19PRC-UKW1/C19PRC-UKW2: The original nine-item IWAH was adapted for use in this study (reference to ‘Americans’ in the original study was substituted with ‘the UK’). Respondents were asked to report on three statements with reference to three groups – people in my community; people from the UK; and all humans everywhere. The three statements were presented to respondents, separately for each of the three groups, as follows: (1) How much do you identify with (feel a part of, feel love toward, have concern for)…? (2) How much would you say you care (feel upset, want to help) when bad things happen to …? And (3) When they are in need, how much do you want to help…? Response scale ranged from 1 ‘not at all’ to 5 ‘very much’.

*2.2.13.6 Attitude towards migrants*. C19PRC-UKW1: Three items from the British Social Attitudes Survey 2015 (British Social Attitudes Survey 2015, 2015) were used to assess respondents’ attitudes towards migrants, as follows: (1) would you say it is generally bad or good for Britain's economy that migrants come to Britain from other countries? (scored on a 10-point scale ranging from 1 ‘extremely bad’ to 10 ‘extremely good’) (2) would you say that Britain's cultural life is generally undermined or enriched by migrants coming to live here from other countries? (scored on a 10-point scale ranging from 1 ‘undermined’ to 10 ‘enriched’); and (3) Some migrants make use of Britain's schools, increasing the demand on them. However, many migrants also pay taxes which support schools and some also work in schools. Do you think that, on balance, migration to Britain reduces or increases pressure on the schools across the whole of Britain (scored on a 5-point scale ranging from 1 ‘reduces pressure a lot’ to 5 ‘increases pressure a lot’)?

*2.2.13.7 Economic individualism and humanitarianism.* C19PRC-UKW2: Items from the American National Election Studies (ANES) Panel Study 2008-2009 (Inter-university Consortium for Political and Social Research, 2010) were adapted to measure economic individualism and humanitarianism in this wave. Respondents were asked indicate the extent to which they agreed with statements adapted from the American National Election Studies (ANES) Panel Study 2008-2009, which were used to assess their level of *economic individualism* (measured by two items ‘hard work offers little guarantee of success’ and ‘any person who is willing to work hard has a good chance of succeeding’) and *humanitarianism* (measured by four items such as ‘everybody in this world has a responsibility to help others when they need assistance’ and ‘assisting people in trouble is not that important to me personally’). All items were scored using a 5-point Likert scale ranging from 1 ‘Strongly disagree’ to 5 ‘Strongly agree’.

*2.2.13.8 Voting behaviour and political party affiliation*. C19PRC-UKW1: Three questions were used to assess respondents’ voting behaviour in the last general election (December 2019) and in the European Referendum (May 2016). All respondents were asked if they had voted (responses voted; did not vote; ineligible to vote, too young; ineligible to vote, not a UK citizen or resident). For those respondents who did vote, in relation to the General Election, they were asked to report which political party they voted for (all main political parties and an ‘other’ option were presented); in relation to the European Referendum, they were asked whether they had voted to ‘Leave’ or ‘Remain in’ the EU. Three additional questions, adapted from the British Election Study 2017 (2017), asked respondents how they would describe their (1) political affiliation (on a 10-point scale ranging from 1 ‘left-wing’ to 10 ‘right-wing’); (2) views on social issues such as abortion and same-sex marriage (on a 10-point scale from 1 ‘very liberal’ to 10 ‘very conservative’); and (3)views on economic issues such as taxes and government spending (on a 10-point scale from 1 ‘very liberal’ to 10 ‘very conservative’).

*2.2.13.9 Preference for news source*. C19PRC-UKW2: Respondents were asked to indicate, from a list of the mainstream newspapers, where they prefer to source news (either in print or online), as a proxy measure for quality of news sourced and political affiliation.

**2.2.14 Trust.**

C19PRC-UKW1/C19PRC-UKW2: Respondents were asked to indicate the extent to which they agree with the statement “*Generally speaking, would you say that most people can be trusted or that you need to be very careful in dealing with people*?” (responses scored on 5-point Likert scale ranging from 1 ‘Most people can be trusted’ to 5 ‘Need to be very careful’). Respondents were asked the extent to which they have trust in the following institutions/groups: (1) parliament; (2) the government; (3) the police; (4) the legal system; (5) political parties; (6) scientists; (7) doctors and other health professionals and (8) pharmaceutical companies. Responses were scored on a 5-point Likert scale ranging from 1 ‘completely trust’ to 5 ‘do not trust at all’.

**2.2.15 Belongingness in neighbourhood**.

C19PRC-UKW1/C19PRC-UKW2: Three questions taken from the UK Community Liver Survey (Cabinet Office, 2015) were asked of respondents to assess their level of belongingness and connectedness to their neighbourhood generally and neighbours specifically, as follows: (1) How strongly do you feel you belong to your immediate neighbourhood? (scored on a 4-point scale from 1 ‘not at all’ to 4 ‘very strongly’; (2) How comfortable would you be with asking a neighbour to keep a set of keys to your home for emergencies (scored on a 4-point scale ranging from 1 ‘very uncomfortable’ to 4 ‘very comfortable’); and (3) How comfortable would you be asking a neighbour to collect a few shopping essentials for you, if you were ill and at home on your own (scored on a 4-point scale ranging from 1 ‘very uncomfortable’ to 4 ‘very comfortable’).

**2.2.16 Religious identity and beliefs.**

C19PRC-UKW1: An 8-item Monotheist and Atheist Beliefs Scale derived from a longer scale developed by the present authors (Alsuhibani, Shevlin, & Bentall, (in submission)) was included. This had four items measuring religiosity, for example ‘The soul is immortal’ and four measuring atheism, for example, “It is wrong to indoctrinate children into religion’. Factor analysis of the longer scale indicated that religiosity and atheism are separate albeit negatively correlated constructs.

**References**

Alsuhibani, A., Shevlin, M., & Bentall, R. P. ((in submission)). Atheism is not the absence of religion: Development of the Monotheist and Atheist Belief Scales and associations with death anxiety and analytic thinking. .

American Psychiatric Association. (2000). *Diagnostic criteria from dsM-iV-tr*: American Psychiatric Pub.

Australian Government Department of Health. (2020). COVID-19: Identifying the symptoms. Retrieved from <https://www.health.gov.au/sites/default/files/documents/2020/03/coronavirus-covid-19-identifying-the-symptoms.pdf>

Bartholomew, K., & Horowitz, L. M. (1991). Attachment styles among young adults: a test of a four-category model. *Journal of personality and social psychology, 61*(2), 226.

Ben‐Ezra, M., Karatzias, T., Hyland, P., Brewin, C. R., Cloitre, M., Bisson, J. I., . . . Shevlin, M. (2018). Posttraumatic stress disorder (PTSD) and complex PTSD (CPTSD) as per ICD‐11 proposals: A population study in Israel. *Depression and anxiety, 35*(3), 264-274.

Birrell, J., Meares, K., Wilkinson, A., & Freeston, M. (2011). Toward a definition of intolerance of uncertainty: A review of factor analytical studies of the Intolerance of Uncertainty Scale. *Clinical Psychology Review*.

Bizumic, B., & Duckitt, J. (2018). Investigating Right Wing Authoritarianism with a Very Short Authoritarianism scale. *Journal of Social and Political Psychology*.

British Election Study 2017. (2017). *British Election Study 2017 Face-to-face survey v1.0 Release note* Retrieved from <https://www.britishelectionstudy.com/wp-content/uploads/2019/01/BES-2017-F2F-codebook.pdf>

British Social Attitudes Survey 2015. (2015). Questionnaire. Retrieved from <http://doc.ukdataservice.ac.uk/doc/8116/mrdoc/pdf/8116_bsa2015_documentation.pdf>

Buhr, K., & Dugas, M. J. (2002). The intolerance of uncertainty scale: Psychometric properties of the English version. *Behaviour research and therapy, 40*(8), 931-945.

Bush, K., Kivlahan, D. R., McDonell, M. B., Fihn, S. D., & Bradley, K. A. (1998). The AUDIT alcohol consumption questions (AUDIT-C): an effective brief screening test for problem drinking. *Archives of internal medicine, 158*(16), 1789-1795.

Cabinet Office. (2015). *Community Life Survey Technical Report 2014-15.* Retrieved from <https://assets.publishing.service.gov.uk/government/uploads/system/uploads/attachment_data/file/470407/Community_Life_2014-15_Combined_technical_report_FINAL.pdf>

Centre for Disease Control and Prevention. (2020a). Coronavirus Disease 2019 (COVID-19): Stop the Spread of Rumous. Retrieved from <https://www.cdc.gov/coronavirus/2019-ncov/symptoms-testing/share-facts.html>

Centre for Disease Control and Prevention. (2020b). Coronavirus Disease (2019): How to protect yourself and others. Retrieved from <https://www.cdc.gov/coronavirus/2019-ncov/prevent-getting-sick/prevention.html?CDC_AA_refVal=https%3A%2F%2Fwww.cdc.gov%2Fcoronavirus%2F2019-ncov%2Fprepare%2Fprevention.html>

Centre for Disease Control and Prevention. (2020c). Corovavirus Disease 2019: How it spreads. Retrieved from <https://www.cdc.gov/coronavirus/2019-ncov/prepare/transmission.html>

Cloitre, M., Hyland, P., Bisson, J. I., Brewin, C. R., Roberts, N., Karatzias, T., & Shevlin, M. (2019). ICD-11 PTSD and complex PTSD in the United States: a population-based study. *Journal of Traumatic Stress*.

Cloitre, M., Shevlin, M., Brewin, C. R., Bisson, J. I., Roberts, N. P., Maercker, A., . . . Hyland, P. (2018). The International Trauma Questionnaire: development of a self‐report measure of ICD‐11 PTSD and complex PTSD. *Acta Psychiatrica Scandinavica, 138*(6), 536-546.

Cohrs, J. C., Maes, J., Moschner, B., & Kielmann, S. (2007). Determinants of human rights attitudes and behavior: A comparison and integration of psychological perspectives. *Political Psychology, 28*(4), 441-469.

Cooper, M. L., Russell, M., Skinner, J. B., & Windle, M. (1992). Development and validation of a three-dimensional measure of drinking motives. *Psychological assessment, 4*(2), 123.

Davidov, E. (2011). Nationalism and constructive patriotism: A longitudinal test of comparability in 22 countries with the ISSP. *International Journal of Public Opinion Research, 23*(1), 88-103.

Davis, M. H. (1980). *Interpersonal reactivity index*: Edwin Mellen Press.

Davis, M. H. (1983). Measuring individual differences in empathy: Evidence for a multidimensional approach. *Journal of personality and social psychology, 44*(1), 113.

Dawson, D. A., Grant, B. F., Stinson, F. S., & Zhou, Y. (2005). Effectiveness of the derived Alcohol Use Disorders Identification Test (AUDIT‐C) in screening for alcohol use disorders and risk drinking in the US general population. *Alcoholism: Clinical and Experimental Research, 29*(5), 844-854.

de Vroege, L., Hoedeman, R., Nuyen, J., Sijtsma, K., & van der Feltz-Cornelis, C. M. (2012). Validation of the PHQ-15 for somatoform disorder in the occupational health care setting. *Journal of occupational rehabilitation, 22*(1), 51-58.

Duckitt, J. (2001). A dual-process cognitive-motivational theory of ideology and prejudice. In *Advances in experimental social psychology* (Vol. 33, pp. 41-113): Elsevier.

Duckitt, J. (2009). Authoritarianism and dogmatism. *Handbook of individual differences in social behavior, 298317*.

Elahi, A., Algorta, G. P., Varese, F., McIntyre, J., & Bentall, R. (2017). Do paranoid delusions exist on a continuum with subclinical paranoia? A multi-method taxometric study. *Schizophrenia Research, 190*, 77-81.

Espie, C. A., Farias Machado, P., Carl, J. R., Kyle, S. D., Cape, J., Siriwardena, A. N., & Luik, A. I. (2018). The Sleep Condition Indicator: reference values derived from a sample of 200 000 adults. *Journal of sleep research, 27*(3), e12643.

Foa, E. B., Huppert, J. D., Leiberg, S., Langner, R., Kichic, R., Hajcak, G., & Salkovskis, P. M. (2002). The Obsessive-Compulsive Inventory: development and validation of a short version. *Psychological assessment, 14*(4), 485.

Fok, C. C. T., Allen, J., Henry, D., & Team, P. A. (2014). The Brief Family Relationship Scale: A brief measure of the relationship dimension in family functioning. *Assessment, 21*(1), 67-72.

Frederick, S. (2005). Cognitive reflection and decision making. *Journal of Economic perspectives, 19*(4), 25-42.

Gilson, K.-M., Bryant, C., Bei, B., Komiti, A., Jackson, H., & Judd, F. (2013). Validation of the Drinking Motives Questionnaire (DMQ) in older adults. *Addictive behaviors, 38*(5), 2196-2202.

Ho, A. K., Sidanius, J., Kteily, N., Sheehy-Skeffington, J., Pratto, F., Henkel, K. E., . . . Stewart, A. L. (2015). The nature of social dominance orientation: Theorizing and measuring preferences for intergroup inequality using the new SDO₇ scale. *Journal of personality and social psychology, 109*(6), 1003.

Horne, Z., Powell, D., Hummel, J. E., & Holyoak, K. J. (2015). Countering antivaccination attitudes. *Proceedings of the National Academy of Sciences, 112*(33), 10321-10324.

Hughes, M. E., Waite, L. J., Hawkley, L. C., & Cacioppo, J. T. (2004). A short scale for measuring loneliness in large surveys: Results from two population-based studies. *Research on aging, 26*(6), 655-672.

Hyland, P., Shevlin, M., Brewin, C. R., Cloitre, M., Downes, A., Jumbe, S., . . . Roberts, N. (2017). Validation of post‐traumatic stress disorder (PTSD) and complex PTSD using the International Trauma Questionnaire. *Acta Psychiatrica Scandinavica, 136*(3), 313-322.

Imhoff, R., & Bruder, M. (2014). Speaking (un‐) truth to power: Conspiracy mentality as a generalised political attitude. *European Journal of Personality, 28*(1), 25-43.

Inter-university Consortium for Political and Social Research. (2010). 2008-2009 American National Election Studies (ANES) Panel Study Questionnaires. Retrieved from <https://www.icpsr.umich.edu/cgi-bin/file?comp=none&study=29182&ds=1&file_id=1047859&path>=

Kahneman, D. (2012). Thinking fast and slow (UK edition). In: London: Penguin.

Karatzias, T., Shevlin, M., Fyvie, C., Hyland, P., Efthymiadou, E., Wilson, D., . . . Cloitre, M. (2016). An initial psychometric assessment of an ICD-11 based measure of PTSD and complex PTSD (ICD-TQ): Evidence of construct validity. *Journal of Anxiety Disorders, 44*, 73-79.

Kossowska, M., Trejtowicz, M., de Lemus, S., Bukowski, M., Van Hiel, A., & Goodwin, R. (2011). Relationships between right‐wing authoritarianism, terrorism threat, and attitudes towards restrictions of civil rights: A comparison among four European countries. *British Journal of Psychology, 102*(2), 245-259.

Kroenke, K., Spitzer, R. L., & Williams, J. B. (2001). The PHQ‐9: validity of a brief depression severity measure. *Journal of general internal medicine, 16*(9), 606-613.

Kroenke, K., Spitzer, R. L., & Williams, J. B. (2002). The PHQ-15: validity of a new measure for evaluating the severity of somatic symptoms. *Psychosomatic medicine, 64*(2), 258-266.

Kroenke, K., Spitzer, R. L., Williams, J. B., & Löwe, B. (2010). The patient health questionnaire somatic, anxiety, and depressive symptom scales: a systematic review. *General hospital psychiatry, 32*(4), 345-359.

Kyriazos, T. A., Stalikas, A., Prassa, K., Galanakis, M., Yotsidi, V., & Lakioti, A. (2018). Psychometric Evidence of the Brief Resilience Scale (BRS) and Modeling Distinctiveness of Resilience from Depression and Stress. *Psychology, 9*(7), 1828-1857.

Levenson, H. (1973). Multidimensional locus of control in psychiatric patients. *Journal of consulting and clinical psychology, 41*(3), 397.

Löwe, B., Decker, O., Müller, S., Brähler, E., Schellberg, D., Herzog, W., & Herzberg, P. Y. (2008). Validation and standardization of the Generalized Anxiety Disorder Screener (GAD-7) in the general population. *Medical care*, 266-274.

Martin, L. R., & Petrie, K. J. (2017). Understanding the dimensions of anti-vaccination attitudes: The vaccination attitudes examination (VAX) scale. *Annals of Behavioral Medicine, 51*(5), 652-660.

Martinez, A. P., Agostini, M., Alsuhibani, A., & Bentall, R. P. (in submission). Mistrust and negative self-esteem: Two paths from attachment styles to paranoia.

McFarland, S., Webb, M., & Brown, D. (2012). All humanity is my ingroup: A measure and studies of identification with all humanity. *Journal of personality and social psychology, 103*(5), 830.

McIntyre, J. C., Wickham, S., Barr, B., & Bentall, R. P. (2018). Social identity and psychosis: Associations and psychological mechanisms. *Schizophrenia bulletin, 44*(3), 681-690.

McManus, S., Bebbington, P., Jenkins, R., & Brugha, T. (2016). *Mental health and wellbeing in England: Adult psychiatric morbidity survey 2014.* Retrieved from Leeds:

Melo, S., Corcoran, R., Shryane, N., & Bentall, R. P. (2009). The persecution and deservedness scale. *Psychology and Psychotherapy: Theory, Research and Practice, 82*(3), 247-260.

Michie, S., Atkins, L., & West, R. (2014). The behaviour change wheel: a guide to designing interventions. *Needed: physician leaders, 26*, 146.

Michie, S., Van Stralen, M. M., & West, R. (2011). The behaviour change wheel: a new method for characterising and designing behaviour change interventions. *Implementation science, 6*(1), 42.

Moos, R. H. (1994). *Family environment scale manual*: Consulting Psychologists Press.

NHS. (2020a). Advice for everyone Coronavirus (COVID-19). Retrieved from <https://www.nhs.uk/conditions/coronavirus-covid-19/>

NHS. (2020b). Who's at higher risk from coronavirus. Retrieved from <https://www.nhs.uk/conditions/coronavirus-covid-19/people-at-higher-risk/whos-at-higher-risk-from-coronavirus/>

Ong, L. M., Visser, M. R., Van Zuuren, F. J., Rietbroek, R. C., Lammes, F. B., & De Haes, J. C. (1999). Cancer patients’ coping styles and doctor–patient communication. *Psycho‐Oncology: Journal of the Psychological, Social and Behavioral Dimensions of Cancer, 8*(2), 155-166.

Oosterhof, N. N., & Todorov, A. (2008). The functional basis of face evaluation. *Proceedings of the National Academy of Sciences, 105*(32), 11087-11092.

Pulos, S., Elison, J., & Lennon, R. (2004). The hierarchical structure of the Interpersonal Reactivity Index. *Social behavior and personality, 32*(4), 355-360.

Rammstedt, B., & John, O. P. (2007). Measuring personality in one minute or less: A 10-item short version of the Big Five Inventory in English and German. *Journal of research in Personality, 41*(1), 203-212.

Robins, R. W., Hendin, H. M., & Trzesniewski, K. H. (2001). Measuring global self-esteem: Construct validation of a single-item measure and the Rosenberg Self-Esteem Scale. *Personality and social psychology bulletin, 27*(2), 151-161.

Rodríguez-Rey, R., Alonso-Tapia, J., & Hernansaiz-Garrido, H. (2016). Reliability and validity of the Brief Resilience Scale (BRS) Spanish Version. *Psychological assessment, 28*(5), e101.

Sapp, S. G., & Harrod, W. J. (1993). Reliability and validity of a brief version of Levenson's locus of control scale. *Psychological Reports, 72*(2), 539-550.

Shapiro, G. K., Holding, A., Perez, S., Amsel, R., & Rosberger, Z. (2016). Validation of the vaccine conspiracy beliefs scale. *Papillomavirus research, 2*, 167-172.

Sirota, M., & Juanchich, M. (2018). Effect of response format on cognitive reflection: Validating a two-and four-option multiple choice question version of the Cognitive Reflection Test. *Behavior research methods, 50*(6), 2511-2522.

Smith, B. W., Dalen, J., Wiggins, K., Tooley, E., Christopher, P., & Bernard, J. (2008). The brief resilience scale: assessing the ability to bounce back. *International journal of behavioral medicine, 15*(3), 194-200.

Spitzer, R. L., Kroenke, K., Williams, J. B., & Löwe, B. (2006). A brief measure for assessing generalized anxiety disorder: the GAD-7. *Archives of internal medicine, 166*(10), 1092-1097.

Tomás-Sábado, J., Gómez-Benito, J., & Limonero, J. T. (2005). The death anxiety inventory: A revision. *Psychological Reports, 97*(3), 793-796.

UK Cabinet Office/Department of Education. (2020). Guidance: Critical workers who can access schools or educational settings. Retrieved from <https://www.gov.uk/government/publications/coronavirus-covid-19-maintaining-educational-provision/guidance-for-schools-colleges-and-local-authorities-on-maintaining-educational-provision>

Vallières, F., Ceannt, R., Daccache, F., Abou Daher, R., Sleiman, J., Gilmore, B., . . . Hyland, P. (2018). ICD‐11 PTSD and complex PTSD amongst Syrian refugees in Lebanon: the factor structure and the clinical utility of the International Trauma Questionnaire. *Acta Psychiatrica Scandinavica, 138*(6), 547-557.

van Zuuren, F. J., de Groot, K. I., Mulder, N. L., & Peter, M. (1996). Coping with medical threat: an evaluation of the Threatening Medical Situations Inventory (TMSI). *Personality and Individual Differences, 21*(1), 21-31.

World Health Organisation. (2020a). Health topics: Coronavirus. Retrieved from <https://www.who.int/health-topics/coronavirus#tab=tab_3>

World Health Organisation. (2020b). Q&A on coronaviruses (COVID-19). Retrieved from <https://www.who.int/news-room/q-a-detail/q-a-coronaviruses>

Yaqub, O., Castle-Clarke, S., Sevdalis, N., & Chataway, J. (2014). Attitudes to vaccination: a critical review. *Social science & medicine, 112*, 1-11.
