# Supplementary material for: Monitoring the psychological, social, and economic impact of the COVID‐19 pandemic in the population: Context, design and conduct of the longitudinal COVID‐19 psychological research consortium (C19PRC) study
Source: Int J Methods Psychiatr Res. 2020 Nov 9;30(1):e1861. doi: 10.1002/mpr.1861 (PMC7992290; doi:10.1002/mpr.1861)
Supplement: Supplementary file 2 — Supplementary Material 2 [file MPR-30-e1861-s002.docx]

Supplementary Table 1 Description of the Socio-political Context^1^ of COVID-19 in the UK, including Number of Confirmed Cases of, and Deaths due to, COVID-19 in the UK (December 2019-May2020)

| **Date** | **Key event** | **Number of confirmed** | |
| --- | --- | --- | --- |
|  |  | **Cases** | **Deaths** |
| 31-12-19 | COVID-19 first detected in Wuhan, China. | 0 | 0 |
| 30-1-20 | WHO declares that COVID-19 meets criteria of being a Public Health Emergency of International Concern | 0 | 0 |
| 1-2-20 | UK Chief Medical Officers (CMOs) advise to increase the UK COVID-19 risk level from low to moderate | 2 | 0 |
| 2-2-20 | UK Government launches a public information campaign to advise on how to slow the spread of COVID-19, emphasising the importance of handwashing. | 2 | 0 |
| 10-2-20 | Secretary of State for Health and Social Care (SSHSC) introduces strengthened powers for public health officials to quarantine people against their will, if necessary | 8 | 0 |
| 1-3-20 | COVID-19 cases detected across UK in England, Wales, Scotland, and Northern Ireland. | 35 | 0 |
| 3-3-20 | UK Government (in collaboration with devolved administrations in Wales, Scotland, and Northern Ireland) publishes a coronavirus action plan which sets out a phased response to the virus (including the ‘contain phase’, the ‘delay phase’, a ‘research phase’ through to the ‘mitigate phase’). | 51 | 0 |
| 6-3-20 | UK records its first COVID-19 related death. | 163 | 1 |
| 9-3-20 | UK Prime Minister (PM) chairs an emergency Cabinet Office Briefing Rooms (COBRA) meeting, attended by the First Ministers of Wales, Scotland, and Northern Ireland. PM announces that the UK remains in the first ‘containment’ phase of the outbreak, but that extensive preparations are being made for a move to the ‘delay’ phase. **Lead investigator, Profess Richard Bentall, initiates contact with collaborators and begins planning for the C19PRC Study.** | 319 | 3 |
| 11-3-20 | UK Chancellor announces a £12bn package of measures to support public services, individuals and businesses affected by COVID-19. This includes additional funding for services, statutory sick pay changes, and a temporary increase in the Business Rates retail discount. | 456 | 6 |
| 12-3-20 | UK moves into the delay phase. CMOs raise the COVID-19 risk in the UK from ‘moderate’ to ‘high’. New advice issued instructs UK citizens to self-isolate for 7 days if they develop a high temperature or a new continuous cough. Citizens are also advised not to go to a GP, pharmacy, or hospital and to only phone the emergency NHS helpline (111) if symptoms do not abate or conditions worsen after 7 days. | 590 | 8 |
| 13-3-20 | New regulations come into force across Wales, England, and Scotland that those who are self-isolating due to COVID-19 are deemed to be incapable of working and are entitled to statutory sick pay. | 798 | 11 |
| 15-03-20 | SSHSC warns that over-70s may be asked to “*self-isolate*”, by not leaving their homes, for an extended period “*within weeks*”. | 1,372 | 35 |
| 16-3-20 | PM updates advice and informs public that, if anyone in a household has a new continuous cough or high temperature, the whole household should self-isolate for 14 days. PM also announces that all non-essential contact and unnecessary travel should cease, and that people should start to work from home where possible. UK Government also announces that they “*will no longer be supporting mass gatherings with emergency workers*” and that social distancing is particularly important for those over 70 years old, pregnant women and those with some underlying health conditions | 1,543 | 55 |
| 17-3-20 | UK Government publishes details of the proposed measures to be included in the fast-tracked coronavirus legislation. | 1,960 | 71 |
| 19-3-20 | UK Secretary of State for Education appears before the Children, Young People and Education Committee to discuss the impact of COVID-19 on education, including the cancellation of this summer’s GCSE and A-level exams. The Coronavirus Bill 2019-21 is introduced in the House of Commons. **First soft-launch (piloting) of the C19PRC-UKW1.** | 3,269 | 144 |
| 20-3-20 | PM announces that the Government are “*telling cafes, pubs, bars, restaurants to close tonight” as well as “nightclubs, theatres, cinemas, gyms and leisure centres*”. Chancellor announces the creation of a Coronavirus Job Retention Scheme where any UK employers will be able to contact Her Majesty’s Revenue and Customs (HMRC) for a grant to cover 80% of the salary of retained workers. The Scheme will cover the costs of wages backdated to 1 March 2020 and is initially set up to cover at least 3 months. **Second soft-launch of revisions of the C19PRC-UKW1.** | 3,983 | 171 |
| 21-3-20 | Regulations requiring the closure of businesses selling food or drink for consumption on the premises come into force in England and Wales. | 5,018 | 233 |
| 23-3-20 | **Official launch of C19PRC-UKW1 (09:00).** PM addresses the nation (20:30)– all people are now required to stay at home except for very limited purposes. Non-essential shops and community spaces will close, and gatherings of more than two people in public are prohibited. These measures are enforceable by the police and other relevant authorities. Government also asks ~1.5 million vulnerable people to ‘shield’ at home for 12 weeks. | 6,650 | 335 |
| 27-3-20 | PM tests positive for COVID-19 and is in self-isolation. Globally, the number of confirmed cases of COVID-19 passes 1 million. | 14,543 | 759 |
| 28-3-20 | New regulations come into force in England, Wales, and Scotland which allow statutory sick pay to be paid from the first day of an employee’s absence due to the coronavirus. This includes those who are unable to work due to having coronavirus symptoms and those in the household of someone with the symptoms. **End of fieldwork for C19PRC-UKW1** | 17,089 | 1,019 |
| 29-3-20 | CMOs announce it could be six months before life can return to ‘*normal*’ because social distancing measures will have to be reduced ‘*gradually*’. | 19,522 | 1,228 |
| 31-3-20 | **Initial findings from C19PRC-UKW1, which suggest adults in the public experienced an increase in levels of anxiety and depression in the immediate aftermath of the government’s lockdown announcement on 23-3-20, are released and reported on in the national media.** | 25,150 | 1,1789 |
| 2-4-20 | SSHSC delivers the daily government briefing having returned to work after completing a period of self-isolation for COVID-19. A target of carrying out 100,000 tests a day by the end of the month (encompassing both swab tests and blood tests is set). Following a rapid review of Personal Protective Equipment (PPE) across the UK, new UK-wide guidance is published. Globally, the number of confirmed cases of COVID-19 passes 1 million, with more than 51,000 confirmed deaths. | 33,178 | 2,921 |
| 5-4-20 | PM is admitted to hospital for tests after testing positive for COVID-19 ten days earlier. | 47,806 | 4,934 |
| 6-4-20 | PM is admitted to intensive care in London. It is announced that the First Secretary of State and Secretary of State for Foreign and Commonwealth Affair (FSS) will deputise. | 51,608 | 5,373 |
| 8-4-20 | UK reports its highest daily death rate of 938 deaths in a 24-hour period. News reports that more than 9 million workers are expected to be furloughed under the Government’s Coronavirus Job Retention Scheme, at an estimated cost to the taxpayer of between £30-40bn. UK Chancellor announces £750 million of funding for the charity sector. A share of this funding will be allocated through the Barnett formula to Wales, Scotland, and Northern Ireland. | 60,773 | 7,097 |
| 9-4-20 | FSS reports that the UK is “*starting to see the impact*” of the restrictions but that it is “*too early*” to lift them and urges people to stay indoors over Easter. PM is moved out of intensive care but remains in hospital. | 65,077 | 7,978 |
| 10-4-20 | UK reaches a new record high of 980 recorded daily deaths from COVID-19. This number is higher than any daily maximum recorded in Italy (or any other European country) during the pandemic to date. Number of confirmed COVID-19 deaths worldwide passes 100,000. | 73,758 | 8,958 |
| 12-4-20 | PM is discharged from hospital having recovered from COVID-19. SSHSC announces that the NHS is developing an app which will alert users whenever they have been in contact with someone who is believed to have contracted COVID-19. | 84,279 | 10,612 |
| 15-4-20 | SSHSC announces new guidelines that will allow close family members to see and say goodbye to dying relatives. Globally, the number of confirmed cases of COVID-19 passes 2 million. | 93,873 | 12,107 |
| 16-4-20 | FSS announces that the COVID-19 lockdown measures will remain in place “*for at least the next three weeks*”. New regulations come into force in Wales, England, and Scotland to extend the statutory sick pay to include those who are extremely vulnerable and at high risk of severe illness from the coronavirus and are advised to stay at home for 12 weeks. | 103,093 | 13,729 |
| 17-4-20 | SSHSC confirms coronavirus tests will be rolled out to cover more public service staff (e.g. police officers, fire fighters, and prison staff). Chancellor extends the Coronavirus Job Retention Scheme to the end of June. | 108,692 | 14,576 |
| 20-4-20 | Online applications for the Coronavirus Job Retention Scheme are opened, with 67,000 claims registered in the first 30 minutes. | 124,743 | 16,509 |
| 21-4-20 | SSHSC states the government is "*throwing everything*" at developing a vaccine as he announces £42.5m for clinical trials being conducted by Imperial College London and the University of Oxford. | 129,044 | 17,337 |
| 22-4-20 | *Soft-launch (piloting) of the C19PRC-UKW2 followed by official launch at 16:00.* | 133,495 | 18,100 |
| 23-4-20 | First human trials of a coronavirus vaccine in Europe begin in Oxford. SSHSC states that daily test capacity has reached 51,000. It is announced that all key workers and members of their households are now eligible for COVID-19 tests and will be able to book tests through the government website from the following day. | 138,078 | 18,738 |
| 24-4-20 | It is announced that the UK will host a ‘*global vaccines summit*’ on June 4 to encourage nations to come together to support the development of a global COVID-19 vaccine. | 143,464 | 19,506 |
| 27-4-20 | PM returns to work after recovering from COVID-19. | 152,840 | 20,732 |
| 28-4-20 | Kawaski syndrome, a rare inflammatory condition thought to be linked to COVID-19 in children, is first reported on in the UK. | 157,149 | 21,092 |
| 30-4-20 | PM announces, “*I can confirm today for the first time that we are past the peak of this disease*”. He also confirmed plans to publish a roadmap to explain how schools and workplaces could reopen safely next week. | 171,253 | 26,771 |
| 1-5-20 | **Fieldwork for C19PRC-UKW2 ends.** | 177,454 | 27,510 |

Note. PM=Prime Minister; SSHSC= Secretary of State for Health and Social Care; FFS=First Secretary of State and Secretary of State for Foreign and Commonwealth Affairs.

^1^ Sources: <https://seneddresearch.blog/2020/03/19/covid-19-timeline-welsh-and-uk-governments-response/>

<https://en.wikipedia.org/wiki/Timeline_of_the_COVID-19_pandemic_in_the_United_Kingdom>

<https://www.mims.co.uk/live-updates-coronavirus-covid-19-uk/infections-and-infestations/article/1673649>
